# Intersectional approaches to cognitive aging: a practical guide to modeling heterogeneous trajectories with GLMM-trees

**DOI:** 10.3389/fpsyg.2026.1738928

**Published:** 2026-07-16

**Authors:** Jeongwon Choi, Belinda Homer, Sunmee Kim

**Affiliations:** 1 Department of Psychology and Human Development, Vanderbilt University, Nashville, TN, United States; 2 Department of Psychology, University of Manitoba, Winnipeg, MB, Canada

**Keywords:** cognitive aging, generalized linear mixed model trees (GLMM-trees), health and retirement study (HRS), intersectionality, longitudinal analysis, recursive partitioning, social determinants of health, subgroup detection

## Abstract

Understanding cognitive aging through an intersectional lens requires analytical methods that can flexibly identify subgroup-specific trajectories across multiple social identities. However, traditional longitudinal models often require researchers to specify how these identities interact in advance, limiting their ability to uncover unexpected patterns of heterogeneity. This article presents a step-by-step guide to applying Generalized Linear Mixed Model Trees (GLMM-trees)—a flexible, recursive partitioning method that integrates mixed-effects modeling with decision-tree algorithms—to uncover distinct cognitive aging patterns shaped by intersecting sociodemographic factors. We demonstrate the utility of this method using data from the U.S. Health and Retirement Study (HRS), with cognitive outcomes measured across five waves. The protocol outlines materials, analytic steps, visualization tools, and strategies to avoid overfitting. We illustrate how GLMM-trees can detect previously unobserved subgroups defined by combinations of education, race, gender, and income that differentially influence both baseline cognitive performance and change over time. By enabling data-driven detection of heterogeneity, GLMM-trees offer a powerful tool for researchers seeking to apply intersectional frameworks to aging research and other domains involving complex longitudinal data.

## 1 Introduction

Cognitive decline in late adulthood is characterized by gradual reductions in abilities such as processing speed, episodic memory, concentration, and learning capacity—changes that are widely regarded as part of the normative aging process (Janoutová et al., 2015; Murman, 2015). These cognitive changes are not only a hallmark of aging but also have tangible implications for daily functioning and overall quality of life in older adults (Harada et al., 2013). In addition, cognitive decline has been identified as a predictor of mortality, further underscoring its importance in understanding aging-related processes (McCammon et al., 2023).

However, the trajectory of cognitive decline is not uniform across individuals (Fletcher et al., 2018; Mungas et al., 2010; Wu et al., 2020; Zheng et al., 2023). Substantial variation has been linked to differences in social identities such as gender, race, educational attainment, financial status, and religious involvement (Marmot, 2020; Warner and Brown, 2011). For example, gender differences in cognitive aging are pronounced, with research indicating that women face unique challenges such as increased psychological distress, limited educational opportunities, and accelerated age-related brain atrophy (Hasselgren et al., 2020; Karp et al., 2004; Yuan et al., 2012). Similarly, chronic exposure to social and economic adversity—especially among marginalized populations such as African Americans, women, and individuals living in long-term poverty—has been linked to steeper cognitive decline in later life (Forde et al., 2019; Hale, 2017; Reuser et al., 2011). The role of religious involvement also appears to differ by race and gender, showing protective effects in Black women but negative associations in White individuals (Henderson et al., 2022). Together, these findings underscore that cognitive aging is shaped by the interplay of multiple, intersecting social dimensions.

To address this complexity, an intersectionality framework (Mullings and Schulz, 2006) offers a valuable lens for understanding how intersecting social factors moderate cognitive aging trajectories. This framework emphasizes that social identities do not act independently but interlock, collectively shaping individuals’ unique experiences within overlapping social contexts (Cooper, 2016; Crenshaw, 1989). This approach allows researchers to move beyond additive models and better understand how overlapping forms of structural disadvantage can compound and shape heterogeneous cognitive aging trajectories (Grzanka et al., 2020).

Capturing this complexity, however, presents significant methodological challenges. Modeling cognitive trajectories shaped by multiple, interlocking social identities requires analytic approaches that can accommodate high-dimensional structures and non-additive interactions. Yet, commonly employed statistical models for analyzing temporal cognitive changes—such as growth curve models and their variants or hierarchical linear models (also known as multilevel models, which are specific types of linear mixed models)—require interaction terms to be predefined and included in the model prior to analysis. This prerequisite limits their capacity to detect important, yet previously unexplored, interactions among multiple social identities. An added complication is the underrepresentation of individuals situated at the intersection of multiple marginalized identities (Purdie-Vaughns and Eibach, 2008). When subgroup sizes are small, conventional models may lack the statistical power or flexibility needed to detect meaningful effects (Feng et al., 2017; Jacobucci et al., 2016; Jacobucci and Grimm, 2018).

To address these methodological challenges of modeling cognitive aging with multiple intersecting variables, this paper presents generalized linear mixed model trees (GLMM-trees) (Fokkema et al., 2018, 2021) as an alternative approach for capturing heterogeneous cognitive aging trajectories. GLMM-trees combine the strengths of mixed-effects models for longitudinal data with decision-tree algorithms for identifying subgroups within a dataset. This approach is useful for modeling both fixed and random effects while revealing hidden structure in the data that may result from interactions among covariates.

Rather than requiring predefined interaction terms, GLMM-trees adaptively segment the data based on observed heterogeneous patterns as the analysis progresses. After fitting a global model, the algorithm selects the variable(s) that best explain variability in the model parameters and splits the data at the optimal cut-off. This process is repeated recursively within each subgroup, allowing the model to identify combinations of variables that explain variation in the outcome. All candidate variables are reconsidered at each step, and the same variable can be reused in different parts of the tree if it continues to add explanatory value. For instance, in a cognitive aging context, even if race is used early on to split the data (e.g., white vs. non-white), it can later be selected again within subsets to further refine the analysis (e.g., white further split by gender, and then within the non-white female group, further racial distinctions can be made) if it explains additional variability in cognitive change within these refined subgroups. This approach ensures that important variables are not prematurely excluded from the analysis, allowing for a more nuanced and comprehensive exploration of interactions.

Moreover, unlike many statistical methods that categorize data into subgroups solely based on predefined social identity variables before analysis (e.g., male vs. female, followed by a separate statistical model), GLMM-trees identify heterogeneous subgroups within the context of the specified cognition model, enabling the discovery of interactions that directly explain variability in cognitive decline patterns. Also, the statistical method itself determines which social factors are significant in explaining differences in cognitive aging patterns, facilitating more exploratory analysis and potentially uncovering unexpected dynamics in the interplay of social identities. Therefore, this approach enables a more flexible, efficient, and comprehensive analysis of cognitive aging. Utilizing such a data-driven heterogeneity identification strategy is particularly advantageous when the appropriate interactions for inclusion in a model are unclear, as often encountered in intersectional research. The automated detection of relevant interactions can simplify the modeling process and save time and resources that would otherwise be spent on extensive preprocessing and testing of multiple subgroup configurations manually.

Motivated by this potential, the purpose of this study is to demonstrate how an intersectional, data-driven approach can serve as a useful tool for studying cognitive aging. We introduce the use of GLMM-trees for this purpose, a method that combines multilevel modeling with decision-tree algorithms to identify subgroups within longitudinal data. Using data from the Health and Retirement Study (HRS), we provide a practical guide to applying GLMM-trees and illustrate how this approach can reveal nuanced patterns of cognitive decline shaped by intersecting social factors. Our goal is to present a practical methodological framework for examining the complexity of cognitive aging grounded in intersectionality.

## 2 Materials and methods

### 2.1 Overview of GLMM-trees

#### 2.1.1 Description of the method

Generalized linear mixed model trees (GLMM-trees) combine linear mixed-effects modeling for clustered or longitudinal data with recursive partitioning to identify meaningful subgroups within the data. Originally proposed by Fokkema et al. (2018) to detect treatment-subgroup interactions in nested data structures, the method has been applied more broadly to multilevel and longitudinal settings (Fokkema et al., 2021). GLMM-trees are particularly useful to examine heterogeneity in outcomes such as cognitive aging where aging patterns may differ by demographic or social factors.

GLMM-trees rely on model-based recursive partitioning (MOB; Zeileis et al., 2008; Zeileis and Hothorn, 2022) that alternates between estimating subgroup-specific fixed effects such as intercepts and time slopes within terminal nodes and estimating random effects globally using the entire dataset, with the globally estimated random effects treated as known offsets during the local fixed-effect estimation step. This structure allows the model to identify variation in fixed effects across subgroups while accounting for individual or cluster-level variability through a common random-effects structure. The choice of random effects depends on the study design. In hierarchical cross-sectional data, random intercepts can represent between-cluster variation. In longitudinal data, both random intercepts and random slopes for time are commonly included to capture individual differences in baseline cognition and rates of cognitive change.

GLMM-trees have been applied across psychology, medicine, and the social sciences. Fokkema et al. (2018) identified patient subgroups that differentially benefited from mental health treatments. Their model pinpointed moderators such as age, baseline severity, and socioeconomic status, uncovering distinct response patterns and informing targeted interventions. Chien et al. (2022) analyzed longitudinal data from older adults in Taiwan to identify key predictors of dementia. Their model included fixed effects such as age, gender, and education, alongside partitioning variables like daily living activities and social support. They highlighted functional ability and social engagement as strong indicators of cognitive decline. Asadi et al. (2024) compared several tree-based methods—including decision trees, random forests, GLMM-trees, and generalized mixed-effects random forests—for predicting cardiovascular disease in a rural Iranian cohort with village-level clustering. Their analysis incorporated 38 variables encompassing demographics, lifestyle factors, medical histories, and biomarkers. Their results indicated that tree-based machine learning models capable of accounting for correlated data, such as GLMM-trees, achieved superior predictive performance.

#### 2.1.2 Advantages over traditional models

A central advantage of GLMM-trees is their ability to identify moderators without requiring interaction terms to be specified in advance. A moderator is a variable that changes the strength or direction of predictor-outcome relationships within a statistical model. In many applied contexts—particularly in cognitive aging research, it is often unclear which variables may serve as moderators or how they interact with each other. Rather than imposing interaction structures in advance, GLMM-trees formally test for parameter instability to evaluate whether model parameters (e.g., intercepts or slopes) vary across values of candidate partitioning variables. A split is introduced only when there is statistical evidence that these parameters differ meaningfully across subgroups. Each terminal node therefore represents a subgroup characterized by its own fixed-effect estimates, such as a distinct baseline level or rate of cognitive change, showing how the predictor-outcome relationship shifts across subgroups (Fokkema et al., 2021).

Compared to traditional regression trees, which treat observations as independent and rely on impurity-based splitting rules (e.g., Gini index), GLMM-trees explicitly model cluster-level or within-subject dependence through random effects and base splits on statistically guided tests of parameter instability, leading to more stable subgroup identification, improved predictive performance in hierarchical settings, and lower Type I error rates (Fokkema et al., 2018, 2021). Their estimation strategy is also computationally efficient. Unlike methods such as SEM trees (Brandmaier et al., 2013) or LongCART (Kundu and Harezlak, 2019), which re-estimate the full model at each step, GLMM-trees alternate between partitioning and updating random effects, reducing computation time while maintaining accuracy (Fokkema and Zeileis, 2024). GLMM-trees are also flexible with respect to outcome distributions. Depending on the specified family, they can accommodate continuous, binary, or count outcomes (e.g., family = binomial for binary data; family = poisson for count data) (Fokkema et al., 2021).

#### 2.1.3 Model estimation and implementation

The GLMM-tree methodology is implemented in the R package *glmertree* (Fokkema et al., 2018) using a formula syntax of the form:Outcome ~ predictors | random effects | partitioning variables.

Predictors before the first bar are treated as subgroup-specific fixed effects; this part can include time-varying predictors, offering useful flexibility for longitudinal data. Random effects after the first bar account for clustering or repeated measures which are estimated globally. Partitioning variables after the second bar guide the algorithm in identifying subgroups, with splits made only if they significantly improve model fit. The package also has options to set the outcome distribution family, specify whether to use REML estimation, control tree complexity, and define clustering structures. It also provides functions such as plot() for tree visualization, predict() for predictions, and coef(), fixef(), and ranef() to extract model coefficients. More details about the *glmertree* package can be found at https://cran.r-project.org/web/packages/glmertree/index.html.

Visualization is useful for understanding GLMM-tree results, especially when the analysis involves multiple partitioning variables and produces different estimates for each subgroup. One of the main visual outputs from GLMM-tree analyses is the tree diagram, which shows the structure of the splits. Each internal node is labeled with the variable used for splitting and the condition applied, such as a cutoff value (if continuous) or a category label (if categorical). Terminal nodes represent the final subgroups and can display summary statistics or subgroup-specific fixed-effect estimates.

The plot() function in the *glmertree* package provides several display options. Using which = “all” shows both the tree structure and the random effects. The option which = “tree” displays only the tree, while which = “tree.coef” produces caterpillar plots of the fixed effects for each terminal node. In longitudinal analyses, plotting predicted outcome trajectories by subgroup can show how patterns related to time differ across identified subgroups. Each node corresponds to a fitted sub-model, enabling users to visualize how outcomes evolve over time for different subgroups. Typically, the *x*-axis represents time points, and the y-axis represents predicted outcomes.

Although this paper does not detail these extensions, many packages can facilitate the analysis and presentation of *glmertree* results. *lme4* (Bates et al., 2015) fits mixed-effects models used inside the tree or on nodewise refits. *partykit* (Hothorn and Zeileis, 2015) handles core tree construction and management—fit, print, plot, predict. *broom.mixed* (Bolker and Robinson, 2024) turns *lme4* or nodewise fits into tidy tables. *effects* and *emmeans* provide adjusted predictions and simple contrasts within each node. Together, *glmertree* can be paired with these tools to make results easier to summarize, compare, and present.

#### 2.1.4 Pruning strategies to control overfitting and enhance interpretability

Recursive partitioning in model-based trees can uncover meaningful heterogeneity, but unconstrained tree growth risks fitting noise or sample-specific artifacts rather than stable, generalizable patterns. To address this, pruning strategies are essential for regulating tree complexity, improving generalization, and maintaining interpretability.

In the *glmertree* implementation of MOB, pre-pruning acts as an early-stopping mechanism during tree construction (Fokkema et al., 2018). At each candidate split, parameter instability tests assess whether fixed-effect parameters (and potentially random effects) vary systematically across levels of a partitioning variable. Splits are only accepted if the corresponding *p*-value falls below a user-specified significance threshold (‘alpha’, default 0.05). To account for the simultaneous evaluation of multiple candidate partitioning variables, *p*-values are Bonferroni-corrected by default (Fokkema and Zeileis, 2024). Lowering alpha (e.g., to 0.01) produces more conservative trees with fewer splits, reducing the risk of overfitting—particularly useful in large observational datasets where even minor heterogeneity may yield statistically significant results due to high power (Zeileis et al., 2008; Zeileis and Hothorn, 2022). Additional pre-pruning controls include minsize (minimum number of observations required in a node before attempting a split) and minbucket (minimum number of observations in each resulting child node). The maxdepth parameter further limits the maximum number of levels in the tree. These parameters collectively prevent overly deep or sparse branches.

While pre-pruning is the default and primary strategy in *glmertree,* post-pruning can be applied optionally to further simplify a fully grown tree by removing branches that do not meaningfully improve fit. In model-based tree workflows, this is often done using in-sample information criteria (e.g., AIC or BIC) to penalize added complexity (Zeileis and Hothorn, 2022). Cross-validation-based post-pruning (e.g., evaluating out-of-sample error on held-out data) is not internally supported in the standard *glmertree* functions but can be implemented manually or via extensions for more rigorous validation.

### 2.2 Data source and sample

#### 2.2.1 Health and retirement study (HRS)

We utilized longitudinal data from the Health and Retirement Study (HRS), RAND HRS Longitudinal File 2020 (V2), to examine cognitive aging trajectories and apply GLMM-trees for identifying intersectional subgroup patterns. The HRS dataset is produced and distributed by the University of Michigan with funding from the National Institute on Aging (grant number NIA U01AG009740) and is a publicly available prospective cohort study of older adults in the United States. The study collects data biennially beginning in 1992, with an overall response rate of 82%.

#### 2.2.2 Sample inclusion criteria

While the HRS includes multiple cohorts, we focused on individuals born between 1931 and 1941 (the HRS cohort) to maintain a consistent age range. We analyzed data from this cohort collected between 1996 (Wave 3) and 2004 (Wave 7), excluding Waves 1 and 2 due to changes in cognitive measures data collection and excluding data beyond Wave 7 due to increased instances of incomplete information. The cohort comprised 10,252 unique individuals across five waves (Waves 3 to 7). All relevant demographic variables related to the intersectionality framework were obtained from the RAND HRS Longitudinal File 2020 (V2).

### 2.3 Measures

#### 2.3.1 Cognitive outcomes

The HRS assesses cognitive functions using a range of self-reported tests that encompass episodic memory, mental status, and vocabulary (McArdle et al., 2007). Our study focuses on two primary cognitive measures as outcome variables from the RAND HRS dataset: episodic memory (Y1) and mental status (Y2), which are the main cognition summary indices in the HRS, consistently collected throughout the study’s waves. Vocabulary was excluded due to its inconsistent administration across the survey’s waves; it was administered only to certain age groups (65 and older) or to new participants who had not been included in previous waves (McCammon et al., 2023).

The cognitive outcomes used in this study were obtained from the HRS Cross-Wave Imputation of Cognitive Functioning Measures file. In this dataset, missing cognition scores for eligible self-respondents were imputed using a multivariate regression-based procedure (Health and Retirement Study, 2023). Proxy respondents and individuals not interviewed in a given wave were excluded from the imputation process. Consequently, any remaining missing values represent structurally ineligible cases (e.g., proxy interviews or wave nonresponse) rather than conventional item-level missingness.

#### 2.3.2 Episodic memory (Y1)

Episodic memory (Y1) is crucial for assessing cognitive aging and potential decline, playing a significant role in the evaluation of dementia and other cognitive impairments (Beck et al., 2012; Twamley et al., 2006). Impairments in episodic memory can significantly affect older adults’ daily functioning and social interaction (Klaming et al., 2017). In the HRS, episodic memory is measured through a combined score from immediate and delayed word recall tests, where possible scores range from 0 to 20. Higher scores indicate better memory performance.

#### 2.3.3 Mental status (Y2)

The HRS defines mental status (Y2) as a key dimension of cognitive function alongside episodic memory (McArdle et al., 2007). Mental status reflects crystallized intelligence, which relates to the accumulation of knowledge and experience (Ding et al., 2019) and is characterized by stronger persistence over time compared to episodic memory (Bianconcini and Cagnone, 2021). In the HRS, mental status is evaluated through a variety of cognitive tasks designed to assess knowledge, language abilities, and orientation (McCammon et al., 2023). These include the Serial 7s test, backwards counting, date naming, object naming, and naming the President and Vice President of the United States. The summary score for mental status ranges from 0 to 15, with higher scores indicating better mental status.

#### 2.3.4 Moderator (social identity) variables

The social identity variables included in this analysis were selected based on both their theoretical relevance to cognitive aging and their availability within the HRS dataset. Guided by prior literature emphasizing the intersectional effects of education, gender, race, socioeconomic status, total income, and religion on later-life cognitive outcomes, we incorporated variables that capture these dimensions as potential moderators. These variables reflect enduring social positions and contextual factors that shape individuals’ exposure to structural advantages and disadvantages over the life course. All selected variables were consistently measured across the HRS waves analyzed.

#### 2.3.5 Time-invariant variables

The time-invariant variables include gender, categorized as male or female; racial identity, classified into three groups: White/Caucasian, Black/African American, and Other; education level, presented in five categories descriptively but dichotomized for analyses based on years of schooling (11 years or less vs. more than 11 years) and religious affiliation, grouped into Protestant, Catholic, Jewish, None/No preference, and Other.

#### 2.3.6 Time-varying variables

The time-varying variables include age, recorded at each survey wave, and total income, computed as the sum of total earnings, pensions, social security, and other income sources for participants and their spouses from the prior year. Total income was used on its original scale (in US dollars). Additionally, the poverty threshold was evaluated using a binary indicator that determined whether the household’s adjusted total income was below the U.S. Census Bureau’s poverty guidelines for the preceding year, adjusted for family size and composition.

### 2.4 Analysis procedure

This section outlines the analytical strategy for identifying heterogeneous cognitive trajectories using GLMM-tree models, including pre-analysis procedures and descriptive exploration. The approach proceeds in three main stages: (1) descriptive analysis and visualization to characterize overall trends and potential subgroup heterogeneity in cognitive aging; (2) preliminary modeling of cognitive change over time via linear mixed-effects models; and (3) application of GLMM-tree to uncover subgroups with distinct trajectories while accounting for multilevel structure.

#### 2.4.1 Handling of missing data and final analytic samples

The initial cohort with episodic memory data across the five waves included 10,252 individuals. Examination of missingness patterns revealed substantial attrition: 5,495 participants (53.6%) had complete data across all waves (0% missing), while the remainder exhibited varying degrees of missingness—979 (9.5%) with 20% missing (one wave), 853 (8.3%) with 40% missing (two waves), 732 (7.1%) with 60% missing (three waves), 846 (8.3%) with 80% missing (four waves), and 1,347 (13.1%) with 100% missing (all waves).

The lmertree()/glmertree() functions in the *glmertree* package do not natively handle missing data and apply listwise deletion by default. Researchers must therefore either restrict analysis to complete cases or pursue imputation. Single imputation approaches (e.g., median/mean for numeric variables, mode for categorical) are straightforward but ignore uncertainty. Multiple imputation (MI), such as via the *mice* package in R, can generate multiple imputed datasets, fit *glmertree* to each, and pool results (e.g., tree structures or variable importance measures) using Rubin’s rules.

However, standard MI procedures like MICE rely on the MAR (Missing At Random) assumption. In longitudinal aging studies such as HRS, dropout is frequently associated with the unobserved values themselves (e.g., greater cognitive or functional decline precipitating non-response), implying MNAR (Missing Not at Random) as the more plausible mechanism (McCammon et al., 2023). Under MNAR, MAR-based MI risks systematic distortion of imputed values—often overly optimistic imputations—leading to biased fixed effect estimates, altered splitting variable selection, misguided cutpoints, and potentially incorrect subgroup identification in GLMM-tree. Because GLMM-tree fits separate GLMMs in each terminal node, trajectory distortions in vulnerable subgroups (e.g., those with severe decline) can propagate to the entire tree structure, including random effects.

Given the high missing rates, the likely MNAR nature of the data, and the absence of fully robust methods for MNAR in multilevel/GLMM-tree contexts, complete-case analysis was adopted as the primary approach. This conservative strategy minimizes reliance on untestable assumptions while preserving observed data integrity. Baseline characteristics of the complete-case subsamples were compared to those of the initial cohort to assess potential systematic differences due to attrition (see Supplementary material 1 for details).

For episodic memory analysis, after further excluding 20 participants missing key social identity variables (11 for religion, 9 for poverty level), the final analytic sample comprised 5,475 participants. Similar to episodic memory, the mental status analysis included only participants with complete data across all waves, totaling 1,345 individuals. Excluding four participants who failed to report their religion, the final sample size was 1,341 participants for mental status analysis.

All results presented in subsequent sections pertain exclusively to these final analytic samples (5,475 for episodic memory; 1,341 for mental status) with complete cognitive data across the five waves and non-missing social identity covariates. If imputation were pursued in future extensions, robustness should be verified through sensitivity analyses under MNAR mechanisms (e.g., pattern-mixture models, delta-adjustment, or tipping-point approaches).

#### 2.4.2 Descriptive analysis and visualization

To provide a foundation for interpreting GLMM-tree results, descriptive analyses and visualizations were conducted to reveal overall patterns in cognitive outcomes and to explore potential heterogeneity across social identity variables. Such visualizations serve multiple purposes: they allow assessment of average trajectories (e.g., whether episodic memory or mental status declines linearly or nonlinearly over time) and highlight intersectional differences that may warrant subgroup exploration.

**Figure 1:**
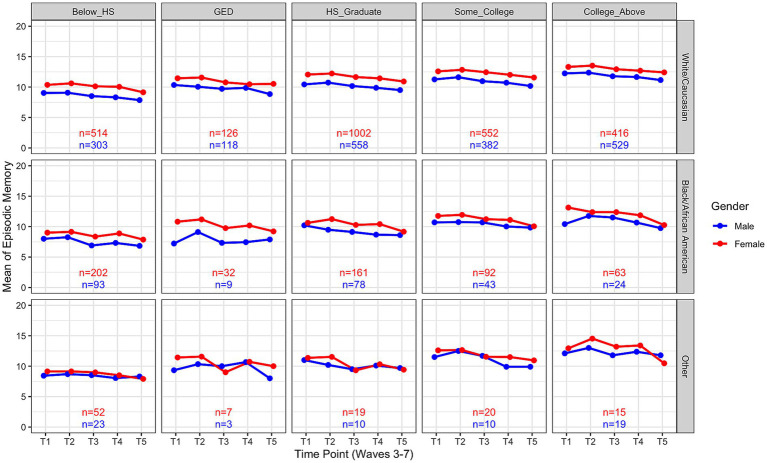
Cognitive aging trends for episodic memory (Y1) across five waves by gender, race, and education level (*N* = 5,475).

**Figure 2:**
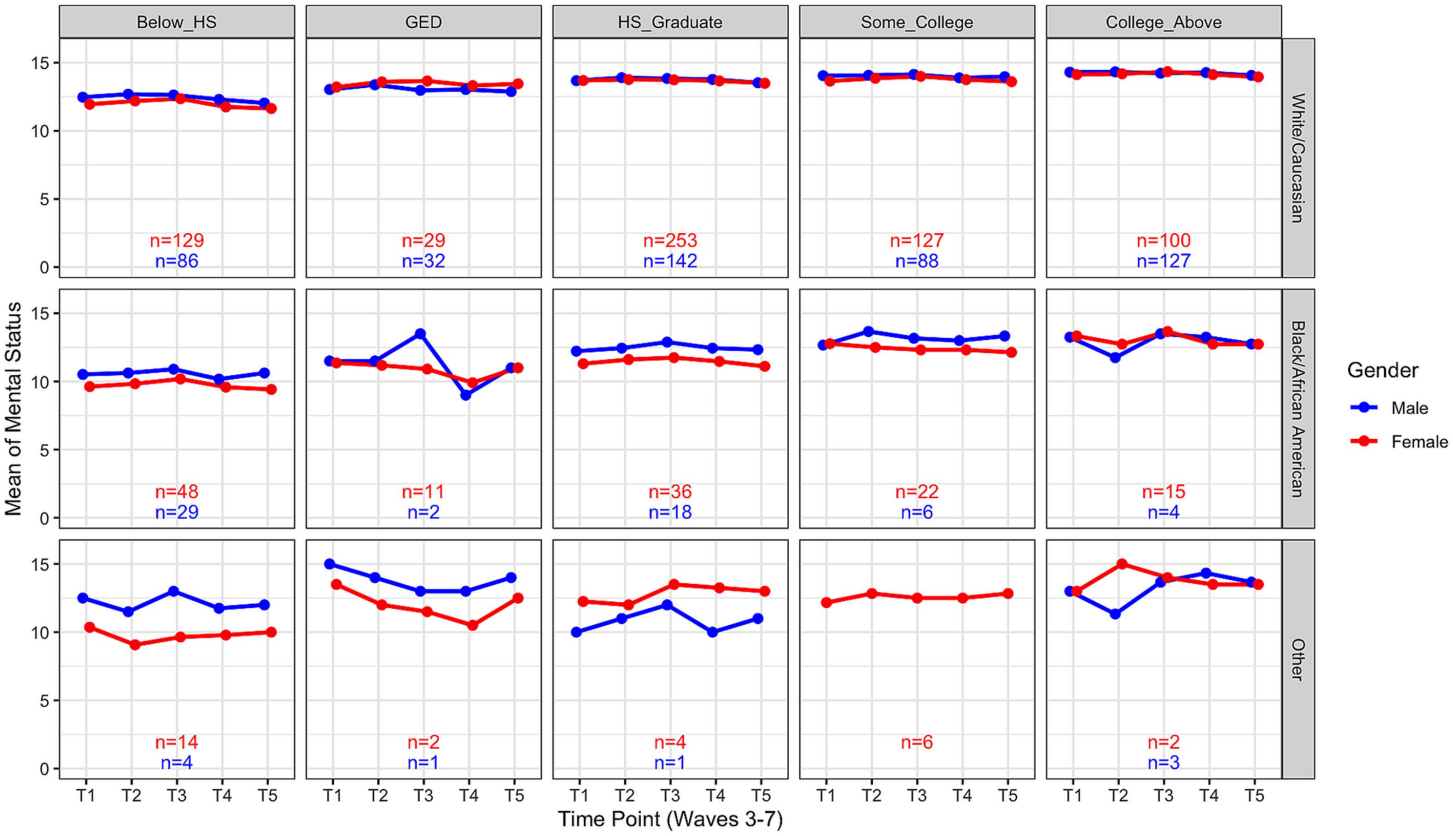
Cognitive aging trends for mental status (Y2) across five waves by gender, race, and education level (*N* = 1,341).

Specifically, cognitive outcome variables (Y1 and Y2) were plotted across the five waves (Wave 3 to Wave 7) to examine temporal trends at the sample level. To investigate intersectionality and potential moderators, trajectories were stratified by combinations of key social identity factors. For instance, line plots displayed cognitive scores over time, with panels arranged by education level (columns), race (rows), and gender differentiated by line color. These figures (Figures 1, 2) illustrate how cognitive aging patterns may vary across demographic subgroups, providing visual evidence of heterogeneity that motivates the use of GLMM-trees for systematic subgroup discovery. Additional stratified plots incorporating other potential moderators are provided in Supplementary material 2 for further reference. Descriptive statistics complemented these visualizations. For continuous variables (including outcomes and time-varying covariates), means and standard deviations were reported; for categorical variables, proportions were presented. Summary statistics appear in Tables 1, 2 (time-varying variables) and Table 3 (time-invariant characteristics).

**Table 2:** Summary statistics for time-varying variables in mental status analysis (*N* = 1,341).

Variable	Statistic	Time points				
		Time 1 (Wave 3)	Time 2 (Wave 4)	Time 3 (Wave 5)	Time 4 (Wave 6)	Time 5 (Wave 7)
Mental status	Mean	13.13	13.23	13.30	13.06	12.94
	SD	2.09	2.13	2.06	2.25	2.30
Age	Mean	63.54	65.40	67.35	69.50	71.46
	SD	1.00	0.98	0.99	0.99	0.99
Total income	Mean	48613.60	46762.49	48501.57	46829.94	46734.82
	SD	65295.14	65023.47	74244.72	75150.28	60098.62
Poverty threshold	% Poverty	0.090	0.087	0.075	0.081	0.067

**Table 3:** Summary statistics for time-invariant variables.

Variable		Episodic memory (*N* = 5,475)		Mental status (*N* = 1,341)	
		*n*	Proportion	*n*	Proportion
Gender	Male	2,202	0.40	543	0.40
	Female	3,273	0.60	798	0.60
Race	White/Caucasian	4,500	0.82	1,113	0.83
	Black/African American	797	0.15	191	0.14
	Other	178	0.03	37	0.03
Education	Below_HS	1,187	0.22	310	0.23
	GED	295	0.05	77	0.06
	HS_Graduate	1,828	0.33	454	0.34
	Some_College	1,099	0.20	249	0.19
	College_Above	1,066	0.19	251	0.19
Religion	Protestant	3,646	0.67	860	0.64
	Catholic	1,454	0.27	391	0.29
	Jewish	90	0.02	23	0.02
	None/NoPref	249	0.05	62	0.05
	Other	36	0.01	5	0.00

**Table 1:** Summary statistics for time-varying variables in episodic memory analysis (*N* = 5,475).

Variable	Statistic	Time points				
		Time 1 (Wave 3)	Time 2 (Wave 4)	Time 3 (Wave 5)	Time 4 (Wave 6)	Time 5 (Wave 7)
Episodic memory	Mean	11.30	11.50	10.91	10.73	10.17
	SD	3.37	3.39	3.36	3.33	3.15
Age	Mean	59.33	61.18	63.14	65.29	67.24
	SD	3.19	3.19	3.18	3.19	3.19
Total income	Mean	57896.44	56924.48	58219.93	57339.64	56605.38
	SD	88372.33	90828.50	110254.27	131689.87	93129.32
Poverty threshold	% Poverty	0.083	0.089	0.088	0.085	0.076

#### 2.4.3 Baseline linear mixed models

To establish a baseline understanding of longitudinal change in cognitive outcomes before partitioning into subgroups, unconditional linear mixed-effects models were fitted separately for episodic memory and mental status. This step serves to quantify average trajectories and individual variability, informing the subsequent GLMM-tree specifications (e.g., inclusion of random slopes where meaningful change is observed).

For episodic memory, which showed a clear linear decline over time in the sample means, a random-intercept and random-slope model was appropriate to capture both between-person differences in baseline performance and individual rates of cognitive change. Person-mean-centered age (age centered within) was included as a time-varying covariate to isolate within-person aging effects from between-person differences in age composition. The model was defined as:lmm_epi <- lmer(episodic_memory ~ age_centered_ / within + (1 + time | participant_id), data = episodic_data)

Here, episodic_memory represents the episodic memory score, time is the wave indicator (centered or coded appropriately), and participant_id accounts for the clustered structure by nesting repeated measures within individuals. This formulation allows for individual differences in baseline performance and the rate of cognitive decline.

In contrast, mental status scores exhibited relatively stable means across waves with minimal systematic within-person change. A simpler random-intercept-only model was therefore sufficient to model between-person variance:lmm_ment <- lmer(mental_status ~ 1 + (1 | participant_id), data = mental_data)

Because no time-varying predictor is included, the subsequent GLMM-tree analysis for mental status identifies subgroup differences in baseline cognitive levels rather than differences in rates of change. These baseline models fitted using the *lme4* package (Bates et al., 2015), provide the random effects structure preserved across terminal nodes in the GLMM-tree analysis, ensuring multilevel accounting while allowing fixed effects to vary by discovered subgroups.

#### 2.4.4 GLMM-tree models

To systematically uncover subgroups with heterogeneous cognitive trajectories while preserving the multilevel structure, GLMM-tree models were fitted using the lmertree() function from the *glmertree* package (Fokkema et al., 2018). Separate models were estimated for each outcome, with the following social identity variables considered as partitioning variables: gender, race, education level, religion, total income, and poverty status.

To balance model flexibility and interpretability while avoiding overfitting—particularly in moderate-sized samples—tree depth was limited to a maximum of 4 levels (via maxdepth = 4), with default values retained for other controls. Because the purpose of this empirical analysis is to illustrate the GLMM-tree framework using the standard implementation provided in R, additional post-pruning procedures—which are typically used to further reduce tree complexity and improve interpretability—were not applied. However, if the resulting tree were considered overly complex, further simplification could be achieved through post-pruning approaches such as information-criterion-based pruning or cross-validation.

The GLMM-tree model was specified as:lt_epi <- lmertree(episodic_memory ~ age_ / centered_within | (1 + time | participant_ / id) | gender + race + education + religion + total_income + poverty, data = episodic_data, maxdepth = 4)

The resulting tree, which displays subgroup-specific fixed effects and trajectories in each terminal node, was generated using:plot(lt_epi, type = “simple”, which = “tree”)

**Figure 3:**
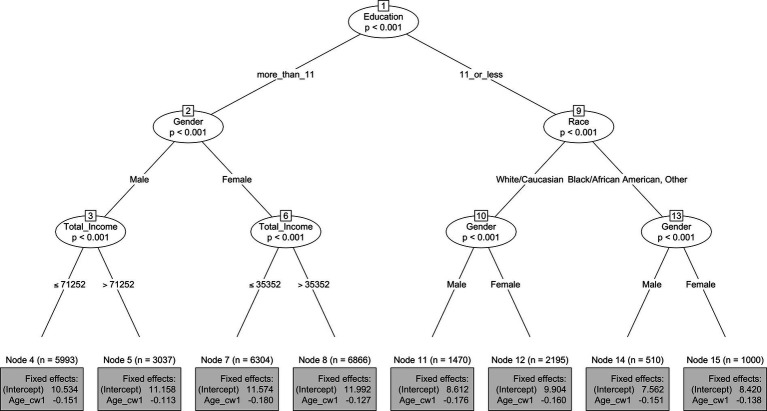
Tree diagram for episodic memory (Y1).

For mental status, a random-intercept-only model was used due to the relatively flat longitudinal pattern. The model was specified as:lt_ment <- lmertree(mental_status ~ 1 | (1 | participant_id) | gender + race + education + religion + total_income + poverty, data = mental_data, maxdepth = 4)

The tree diagram displays the subgroup structure along with boxplots of the observed outcome variable within each terminal node. It was created using the following syntax:plot(lt_ment, which = “tree”, fitted = “combined”)

**Figure 4:**
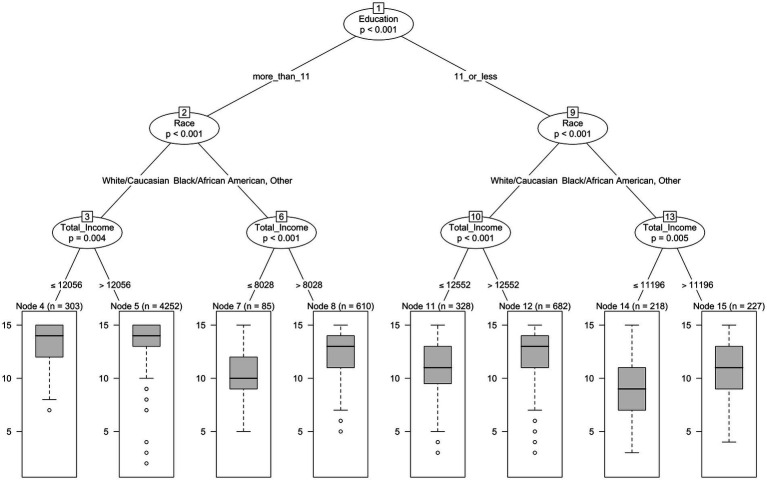
Tree diagram for mental status (Y2).

#### 2.4.5 Software and code reproducibility

All analyses were conducted in R (version 4.3.2) using the *glmertree* package (version 0.2.4; Fokkema et al., 2018), alongside *lme4* for baseline mixed models. Annotated example code for model fitting, tree estimation, and visualization is provided in Supplementary material 3. Annotated scripts are included to support reproducibility and allow researchers to replicate the analysis or adapt it to their own datasets.

## 3 Results

### 3.1 Descriptive statistics

#### 3.1.1 Sample characteristics

Table 3 presents summary statistics for time-invariant variables, including gender, education level, and religion. Demographically, both samples consist of approximately 60% female participants and are predominantly White/Caucasian (82% in Y1, 83% in Y2), with about 15% identifying as Black/African American. Educational levels are similar across both samples, with roughly 22–23% having less than a high school education, 33–34% being high school graduates, and 19% having a college degree or higher. The majority of participants are Protestant (67% in Y1, 64% in Y2), followed by Catholic (27 and 29%, respectively). Overall, the samples exhibit consistent demographic characteristics, with both showing a decline in cognitive function over time, reflecting common aging trends.

#### 3.1.2 Longitudinal patterns in cognitive outcomes

Tables 1 and 2 present summary statistics for the mental status and episodic memory samples, respectively, across the five measurement occasions. The descriptive analysis revealed distinct trends for the two outcome variables. For episodic memory (Y1, N = 5,475), the mean score declined from 11.30 to 10.17 over the five time points, with a standard deviation of 3.3. Participants’ ages range from an average of 59.33 to 67.24 years, with about 8% falling below the poverty threshold and a mean total income ranging from approximately $56,600 to $58,200. For mental status (Y2, N = 1,341), the mean score decreased slightly from 13.13 to 12.94, and the standard deviation rose from 2.1 to 2.3. The participants in this sample are older, with the average age rising from 63.54 to 71.46 years, with the poverty rate declining from 9.0% in Wave 3 to 6.7% in Wave 7, and a mean total income decreasing from about $48,000 to $46,000. The discrepancy in age is attributed to the mental status measurements being conducted on a specific, limited sample based on predefined criteria, resulting in an older cohort with a narrower age range. Overall, the mean episodic memory scores showed a clear downward trend over time, while mental status scores remained relatively stable, suggesting differing trajectories for these two cognitive outcomes.

#### 3.1.3 Subgroup visualizations

Figures 1, 2 display the observed trajectories of episodic memory and mental status over time, with sample sizes for each gender shown in the corresponding panels. Although aggregate trends suggest modest cognitive decline over time, visual inspection of Figures 1 and 2 revealed apparent heterogeneity across subgroups in both baseline cognitive levels and rates of decline. For example, some subgroups began with lower scores or experienced sharper declines over time. These patterns emphasize the importance of analyzing cognitive aging at the subgroup level rather than relying solely on sample-wide averages, which may mask meaningful differences in trajectories across intersecting social identities.

### 3.2 Baseline linear mixed models

#### 3.2.1 Episodic memory

Table 4 presents the results of the linear mixed model for episodic memory. The fixed intercept was 10.92 (SE = 0.03), and the fixed effect of person-mean-centered age was −0.15 (SE = 0.01, *t* = −28.69), indicating a significant within-person decline in episodic memory with aging. The random effects revealed substantial individual differences, with intercept variance at 7.43 and slope variance at 0.08. A negative correlation between intercept and slope (*ρ* = −0.54) suggests that individuals with higher initial memory scores tended to experience faster declines over time. The residual variance was 5.25.

**Table 4:** Linear mixed-effects model results for episodic memory.

Fixed effects	Estimate	SE	*t*
Intercept	10.92	0.03	312.57
Age_centered_within	−0.15	0.01	−28.69

Random effects	Variance	SD	Correlation
Intercept	7.43	2.73	—
Time	0.08	0.29	−0.54
Residual	5.25	2.29	—

#### 3.2.2 Mental status

Table 5 presents the results of the linear mixed model for mental status. The fixed intercept was 13.13 (SE = 0.05), reflecting stable baseline cognitive performance in this sample. Approximately 64% of the total variance was due to between-person differences, with a random intercept variance of 3.04 and a residual variance of 1.68. These baseline findings provide a foundation for the GLMM-tree analyses, which will examine how cognitive patterns differ across subgroups.

**Table 5:** Linear mixed-effects model results for mental status.

Fixed effects	Estimate	SE	*t*
Intercept	13.13	0.05	262

Random effects	Variance	SD
Intercept	3.04	1.74
Residual	1.68	1.29

### 3.3 GLMM-tree results

#### 3.3.1 Episodic memory (Y1) tree model

The results of the episodic memory GLMM-tree model are shown in Figure 3. The fitted model produced a residual sum of squares of 117,143.3. The estimated random intercepts ranged from −7.53 to 6.95, reflecting substantial between-person variability in baseline episodic memory. A convergence warning occurred during model fitting. Such warnings can arise in mixed-effects models when variance–covariance parameters are difficult to estimate precisely, particularly in models that include both random intercepts and random slopes. In this case, the warning suggests that the random-effects variance–covariance components may be estimated with limited precision; therefore, interpretation of the random slope variance and the intercept–slope correlation should be approached with caution. The fixed-effect estimates used to characterize subgroup trajectories remain interpretable.

The tree identified Education, Gender, Race, and Total Income as relevant partitioning variables. Education emerged as the primary splitting variable. Among participants with higher education, additional splits occurred by gender and total income, whereas among participants with lower education the tree further partitioned the sample by race and gender. In total, eight terminal nodes were identified, representing distinct intersectional subgroups.

Among participants with more than 11 years of education, gender and income differentiated cognitive trajectories. Men with higher income (> $71,252) had a baseline episodic memory score of 11.16 and declined by approximately 0.11 points per year of aging (slope = −0.11). Men with lower income (≤ $71,252) had a lower baseline score of 10.53 and experienced a steeper decline of 0.15 points per year (slope = −0.15). Women in this education group showed higher baseline scores overall. Higher-income women (> $35,352) had a baseline score of 11.99 and declined by 0.13 points per year (slope = −0.13), whereas lower-income women began at 11.57 and exhibited the steepest decline in this subgroup, with a slope of −0.18.

Among participants with 11 years of education or less, race and gender further differentiated cognitive trajectories. White/Caucasian men had a baseline episodic memory score of 8.61 and declined by approximately 0.18 points per year (slope = −0.18), whereas White/Caucasian women had a higher baseline score of 9.90 with a slightly slower decline (slope = −0.16). Non-White men exhibited the lowest baseline cognitive performance (7.56) with a slope of −0.15, while non-White women had a baseline score of 8.42 and showed the slowest rate of decline within this education group (slope = −0.14).

Overall, the GLMM-tree results indicate that both baseline cognitive levels and age-related rates of decline vary across intersectional combinations of education, gender, race, and income, illustrating heterogeneity in cognitive aging trajectories that would not be captured by models assuming homogeneous effects across the population.

#### 3.3.2 Mental status (Y2) tree model

The results of the mental status GLMM-tree model are presented in Figure 4. The fitted model yielded a residual sum of squares of 9,384.6. The estimated random intercepts ranged from −5.54 to 3.58, indicating substantial between-person variability in baseline mental status.

Consistent with the episodic memory analysis, Education emerged as the primary partitioning variable, followed by splits on Race and Total Income, resulting in eight terminal nodes. Because the mental status model included only a random intercept, the resulting tree reflects heterogeneity primarily in baseline cognitive performance across social subgroups, rather than differences in rates of cognitive change.

Several notable patterns emerged across the identified subgroups. Within the higher education group, income further differentiated mental status levels within racial groups. For example, among White/Caucasian participants with more than 11 years of education, those with higher income (> $12,056) exhibited the highest observed mental status scores (mean = 13.84), while those with lower income had slightly lower scores (mean = 13.54). A similar income gradient was observed among Black/African American and other racial groups in this education category, where higher-income participants (mean = 12.27) had noticeably higher scores than those with lower income (mean ≈ 11.38).

A particularly pronounced disparity appeared among participants with lower education and minority racial status. In this group, non-White participants with lower income (≤ $11,196) exhibited the lowest mental status scores across all identified subgroups (mean = 9.65). In contrast, White/Caucasian participants with similar educational attainment showed substantially higher cognitive levels, especially among those with higher income (mean = 12.35).

## 4 Discussion

This study demonstrated how GLMM-trees can be applied to examine heterogeneous trajectories of cognitive aging within an intersectionality framework. In longitudinal aging research, it is often unclear which social identities differentiate baseline cognitive levels, which relate to rates of change, and how these identities combine within subgroups. GLMM-trees address this by using statistically guided recursive partitioning to identify subgroup structures, while preserving a mixed-effects framework that accounts for within-person dependence. The resulting tree provides an interpretable representation of how combinations of background characteristics correspond to different cognitive profiles over time.

Despite the increasing availability of large, complex aging datasets, applied examples that systematically integrate exploratory heterogeneity detection with formal longitudinal modeling remain relatively limited. The present study contributes to this gap by demonstrating a concrete, step-by-step workflow based on the publicly available HRS data, with fully reproducible code implemented in the *glmertree* package. In doing so, it illustrates GLMM-trees as a structured analytic process for incorporating intersectionality in longitudinal research: researchers first characterize overall patterns in the data, then anchor the longitudinal structure with an appropriate mixed-effects model, and finally evaluate whether—and where—systematic subgroup differences are supported by statistical evidence. Overall, GLMM-trees represent a promising and interpretable tool for generating nuanced, data-driven insights into intersectional dynamics in cognitive aging.

Analysis of the HRS data revealed that education, gender, race, and income emerged as key moderators of cognitive trajectories. Our findings on these individual moderators align broadly with prior research. For example, higher education has consistently been linked to better baseline episodic memory performance (Lachman et al., 2010; Liampas et al., 2023; Lövdén et al., 2020; Rodriguez et al., 2021), and subgroups with higher education in our tree exhibited elevated baseline scores. Similarly, women frequently exhibit advantages in baseline episodic memory (Asperholm et al., 2019; Bloomberg et al., 2021), a pattern mirrored in female subgroups here. Racial differences also corresponded to established evidence, with White/Caucasian individuals generally demonstrating higher baseline scores relative to other groups (Fyffe et al., 2011; Gross et al., 2015; Stinchcombe and Hammond, 2022).

In terms of longitudinal change, our results contribute to the ongoing discussion about the role of education. Although education is often viewed as protective against cognitive decline, many studies suggest that this relationship is more complex: higher education may be linked to higher initial performance but not necessarily to slower decline (Clouston et al., 2020; Lenehan et al., 2015; Nyberg et al., 2012; Rönnlund et al., 2005; Zahodne et al., 2011). Our GLMM-tree findings are consistent with this interpretation, as education primarily differentiated baseline levels rather than rates of change across subgroups. We also observed that women experienced faster declines in episodic memory in certain subgroups, which aligns with results reported in other longitudinal studies (Jin et al., 2023; Liampas et al., 2023).

Importantly, however, the identified moderators did not operate in isolation. The tree structure revealed that the ordering and interaction of moderators differed across cognitive outcomes, highlighting the value of an intersectional perspective. Education emerged as the primary moderator for both episodic memory and mental status, yet the subsequent sources of heterogeneity diverged across outcomes. For episodic memory, heterogeneity within higher education groups was primarily structured by gender, whereas within lower education groups, race played a more prominent role. In contrast, for mental status, race consistently emerged as the dominant secondary moderator across education levels. These differences suggest that distinct cognitive domains may be shaped by different interplays of social stratification, even when sharing similar high-level predictors.

Several methodological considerations arise from this analysis. A key limitation of this study is the reliance on complete-case analysis in the presence of substantial attrition, particularly given the longitudinal nature of the HRS data and the likelihood that missingness in cognitive measures is not missing at random. Although multiple imputation is commonly used to address missing data, simulation evidence from longitudinal settings indicates that standard MAR-based techniques (such as MICE) often fail to produce unbiased estimates under MNAR mechanisms, even with the inclusion of auxiliary variables (Wijesuriya et al., 2025; Madley-Dowd et al., 2025). By restricting the analytic samples to participants with complete episodic memory (*n* = 5,475) and mental status (*n* = 1,341) data across all five waves, we prioritized avoidance of assumptions that could introduce systematic bias under MNAR mechanisms, but this necessarily reduced sample size and may have limited statistical power and generalizability to the full cohort, especially among subgroups more prone to dropout (e.g., those with steeper cognitive decline). Baseline characteristics of the retained subsamples were reasonably comparable to the initial cohort, yet residual selection effects cannot be fully excluded. Future studies could bolster confidence in these results by incorporating targeted sensitivity analyses under explicit MNAR assumptions (e.g., pattern-mixture models or delta-adjustment methods), extensions that were beyond the primary scope of the present work but remain valuable for evaluating the robustness of heterogeneous cognitive trajectories in aging populations.

Another consideration relates to the representativeness of subgroups identified in the GLMM-tree analyses and the exploratory nature of the method itself. The HRS oversamples Black and Hispanic participants to enhance minority representation, yet the complete-case subsamples remain predominantly non-Hispanic White (reflecting patterns of differential attrition and baseline non-response in longitudinal aging data), which may limit the detection or stability of heterogeneous trajectories among non-White groups. This could result in moderators or subgroups that are less generalizable beyond the retained, more advantaged participants. Furthermore, although tree complexity was constrained to promote interpretability and mitigate overfitting, GLMM-tree remains an exploratory tool prone to sample-specific instability. Additional robustness checks, such as bootstrapping or resampling-based validation of tree structure and cutpoints for splits, would strengthen confidence in the identified moderators but were not feasible within the scope of this study.

Overall, these findings underscore two complementary insights. Substantively, cognitive aging trajectories are structured by layered and intersecting social positions—most prominently anchored by education—rather than by single demographic characteristics. Methodologically, GLMM-trees provide a transparent and statistically principled approach for investigating such heterogeneity in longitudinal data, offering a more nuanced understanding of inequality in aging.

## Data Availability

Publicly available datasets were analyzed in this study. The datasets analyzed in this study were obtained from the Health and Retirement Study (HRS), including the RAND HRS Longitudinal File 2020 (V2) and the HRS Cross-Wave Imputation of Cognitive Functioning Measures 1992–2020 file. Public-use datasets can be accessed at: https://hrs.isr.umich.edu/data-products.
