# Supplementary material for: Intersectional approaches to cognitive aging: a practical guide to modeling heterogeneous trajectories with GLMM-trees
Source: Front Psychol. 2026 Jul 16;17:1738928. doi: 10.3389/fpsyg.2026.1738928 (PMC13421182; doi:10.3389/fpsyg.2026.1738928)
Supplement: Supplementary file 1 [file Supplementary_file_1.docx]

Supplementary Material

# Assessing the Impact of Missing Data on Key Variable Distributions

This section aims to ascertain the robustness and validity of our findings by examining the distribution patterns of key variables – gender, race, education, religion, and total income. For simplicity, we included only the Total Income variable to reflect personal financial situation, as it provides more detailed information compared to poverty thresholds. This examination involves contrasting these distributions in the original dataset against those in a subset where no missing values are present for cognitive assessments, Episodic Memory (Y1) and Mental Status (Y2). The provided plots demonstrate the similarity in distributions (by comparing the density of observations) for these key variables, indicating the subset's representativeness, thereby mitigating concerns about potential biases introduced due to missing data. This strategy is particularly pertinent considering the existing research gap in managing missing data within the context of multilevel models and generalized linear mixed model trees (GLMM-trees). Consequently, this comparative approach was deemed most appropriate for our analysis.

## Supplementary Figures


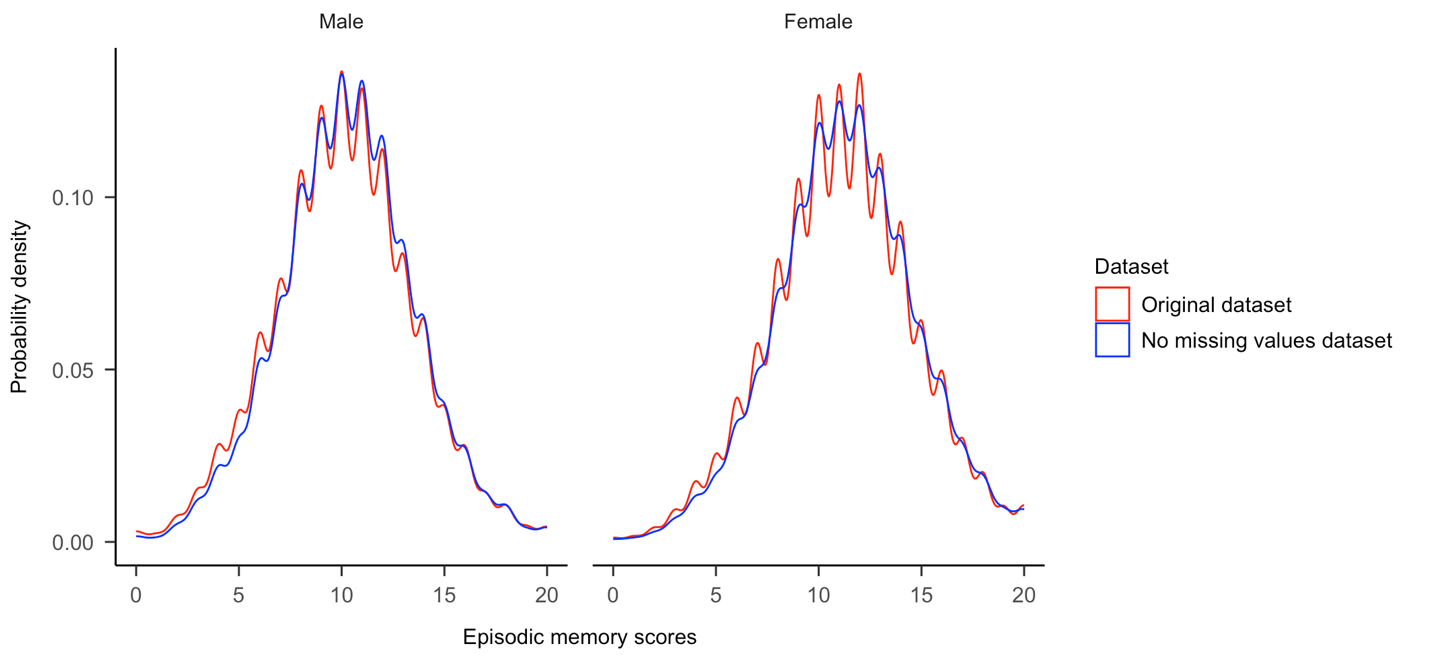
**Supplementary Figure 1.** Comparison of Episodic Memory (Y1) by Gender Between Original and No Missing Data Subsets


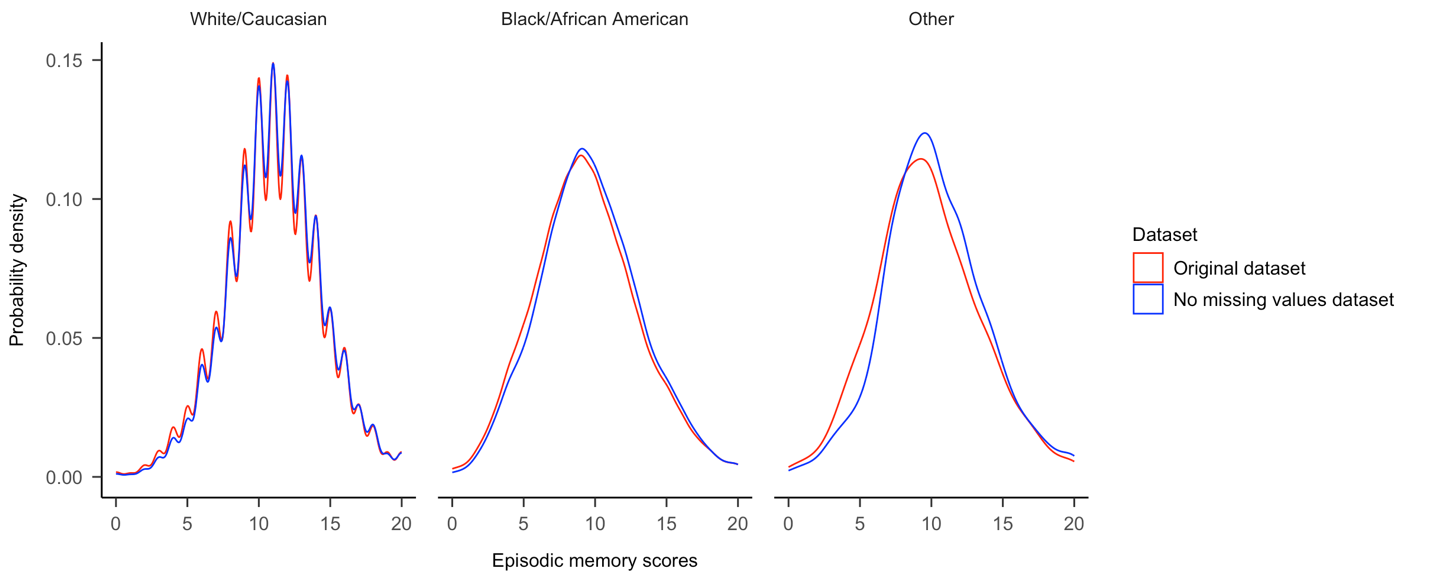


**Supplementary Figure 2.** Episodic Memory (Y1) Distribution by Race in Original and No Missing Data Subsets


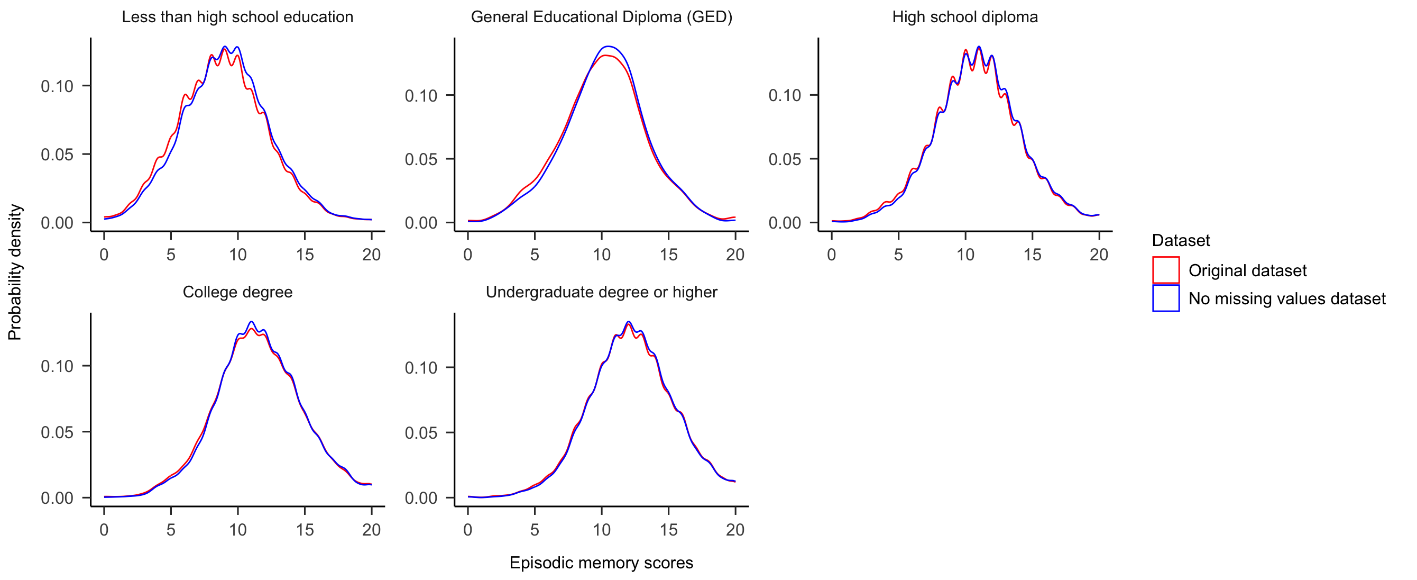


**Supplementary Figure 3.** Educational Impact on Episodic Memory (Y1) in Original and No Missing Data Sets


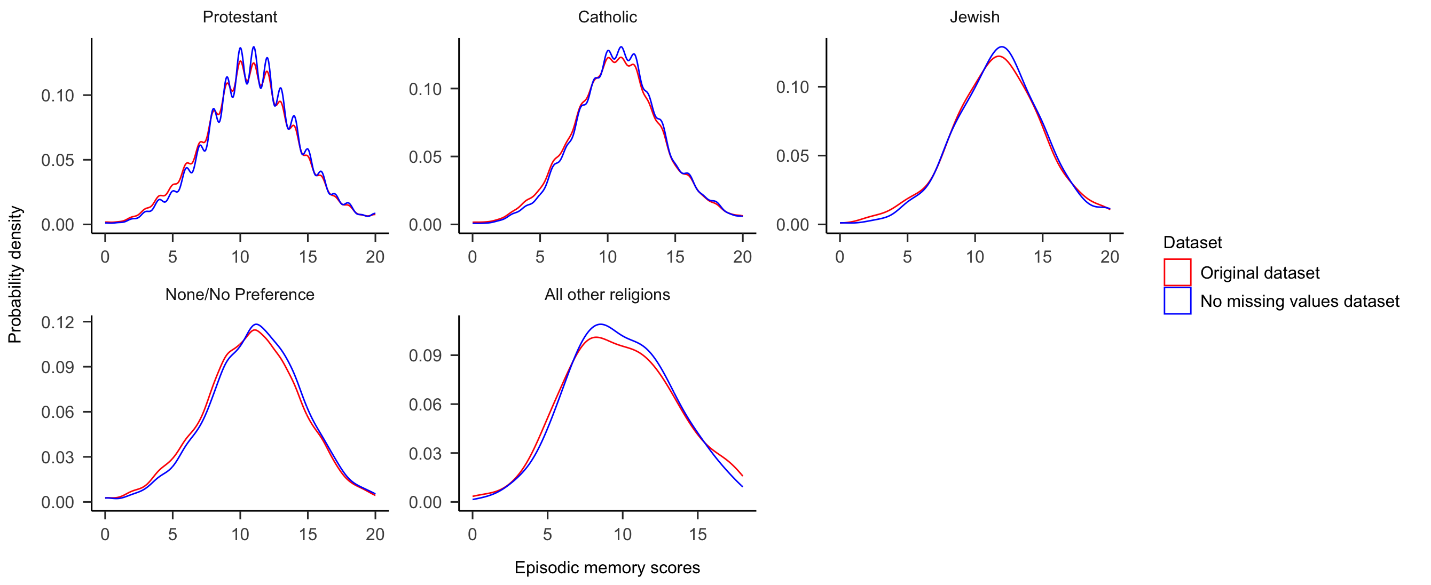


**Supplementary Figure 4.** Distribution of Episodic Memory (Y1) by Religion in Original and No Missing Data Subsets

*
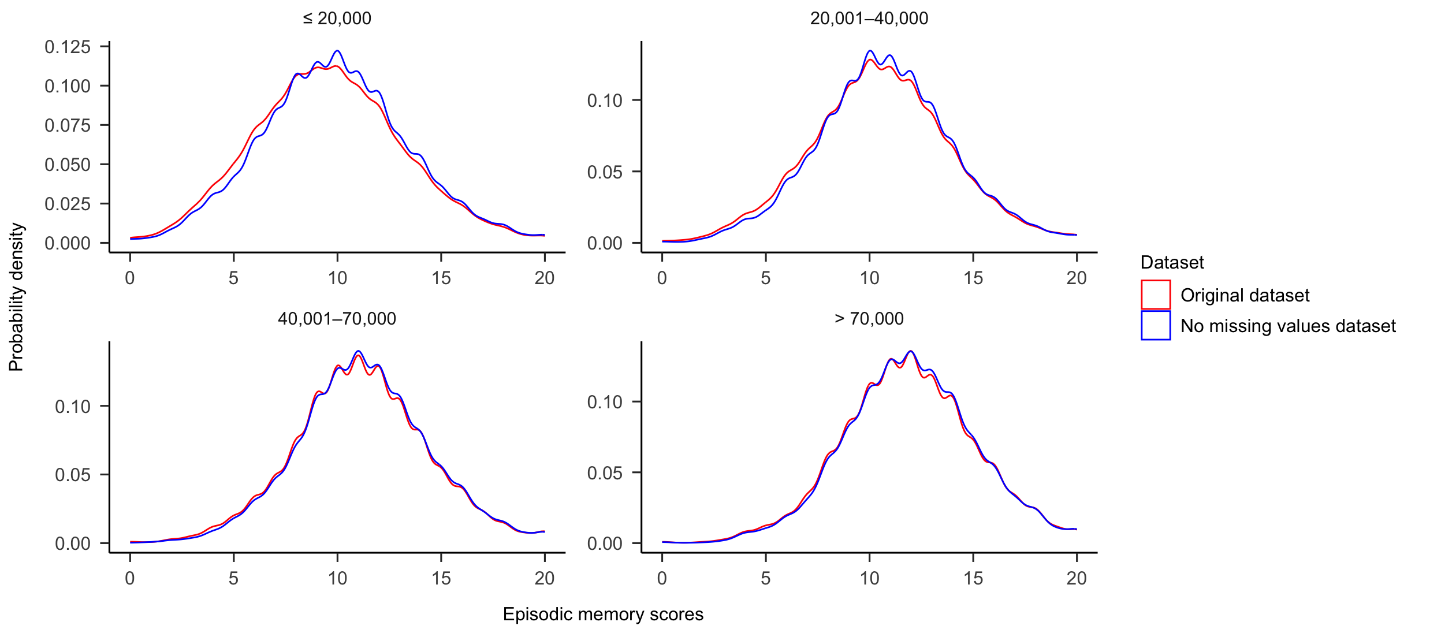
*

**Supplementary Figure 5.** Distribution of Episodic Memory (Y1) by Income Level in Original and No Missing Data Subsets

*Note.* Data for income is in U.S. dollars per annum.

**
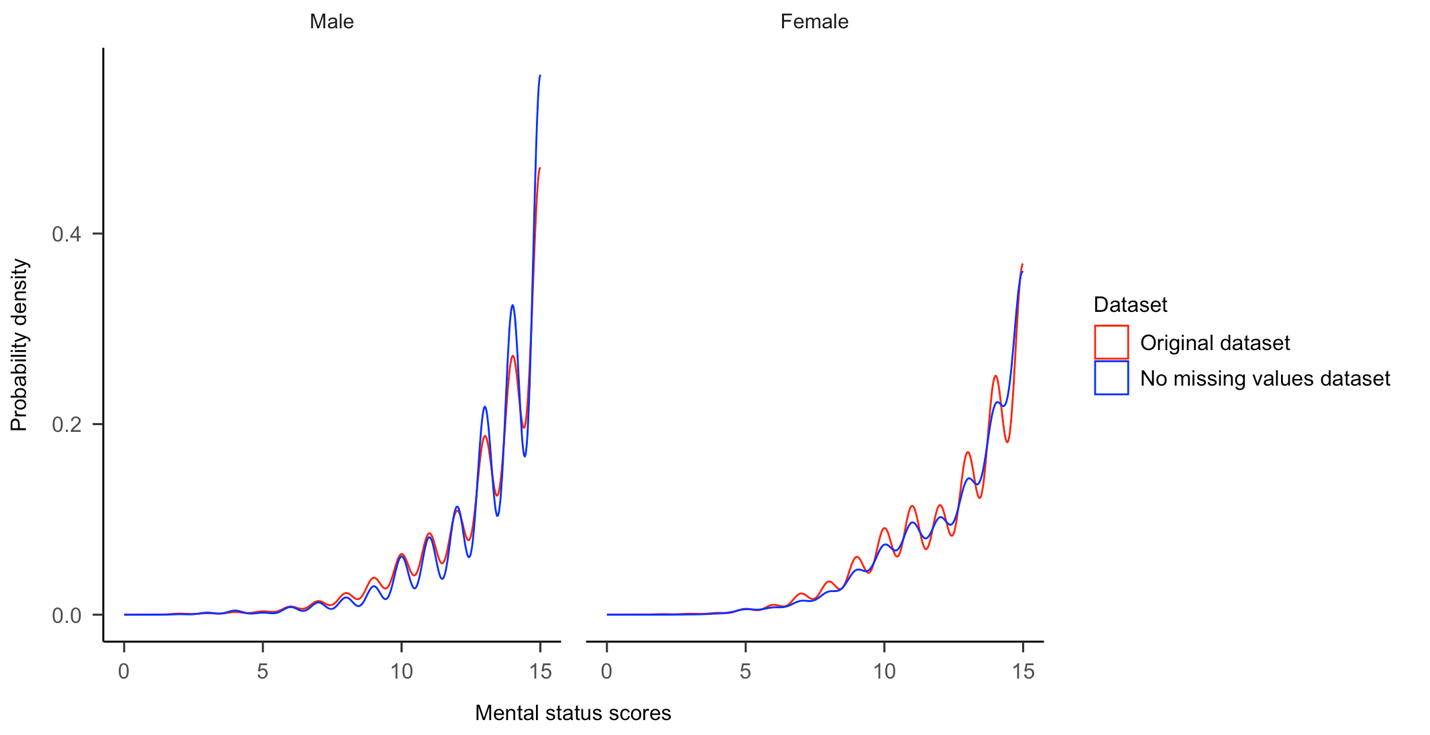
**

**Supplementary Figure 6.** Comparison of Mental Status (Y2) by Gender Between Original and No Missing Data Subsets

**
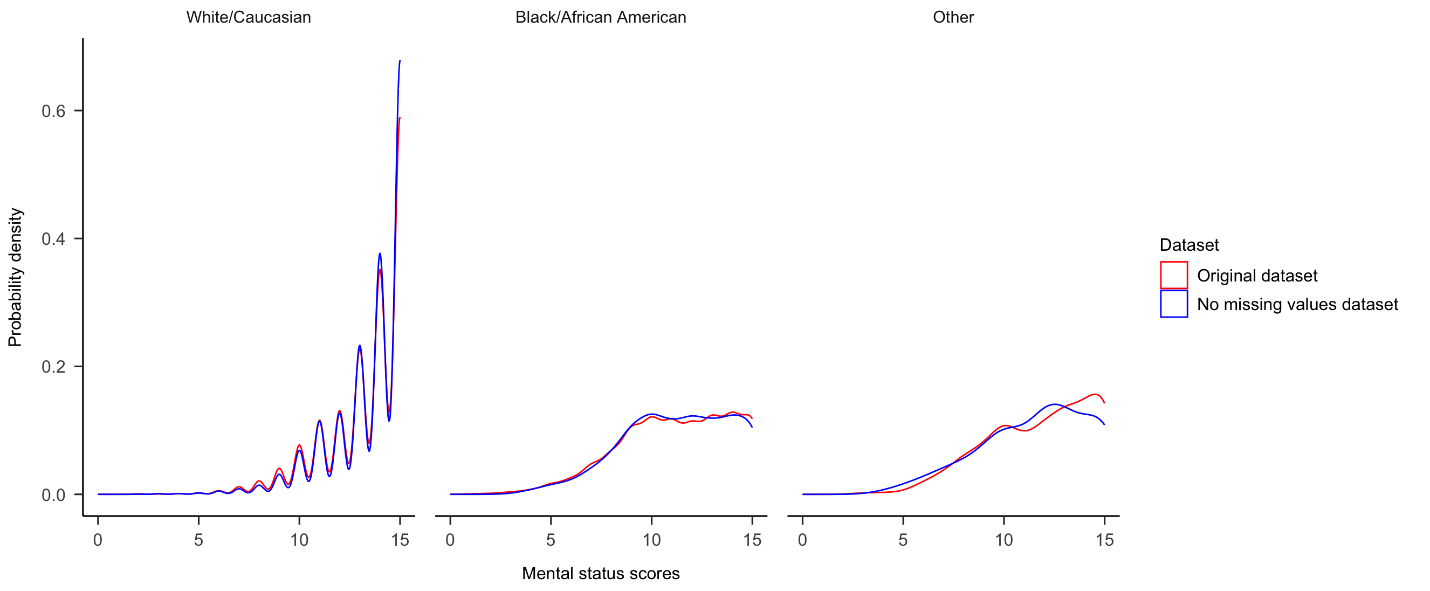
**

**Supplementary Figure 7.** Mental Status (Y2) Distribution by Race in Original and No Missing Data Subsets

**
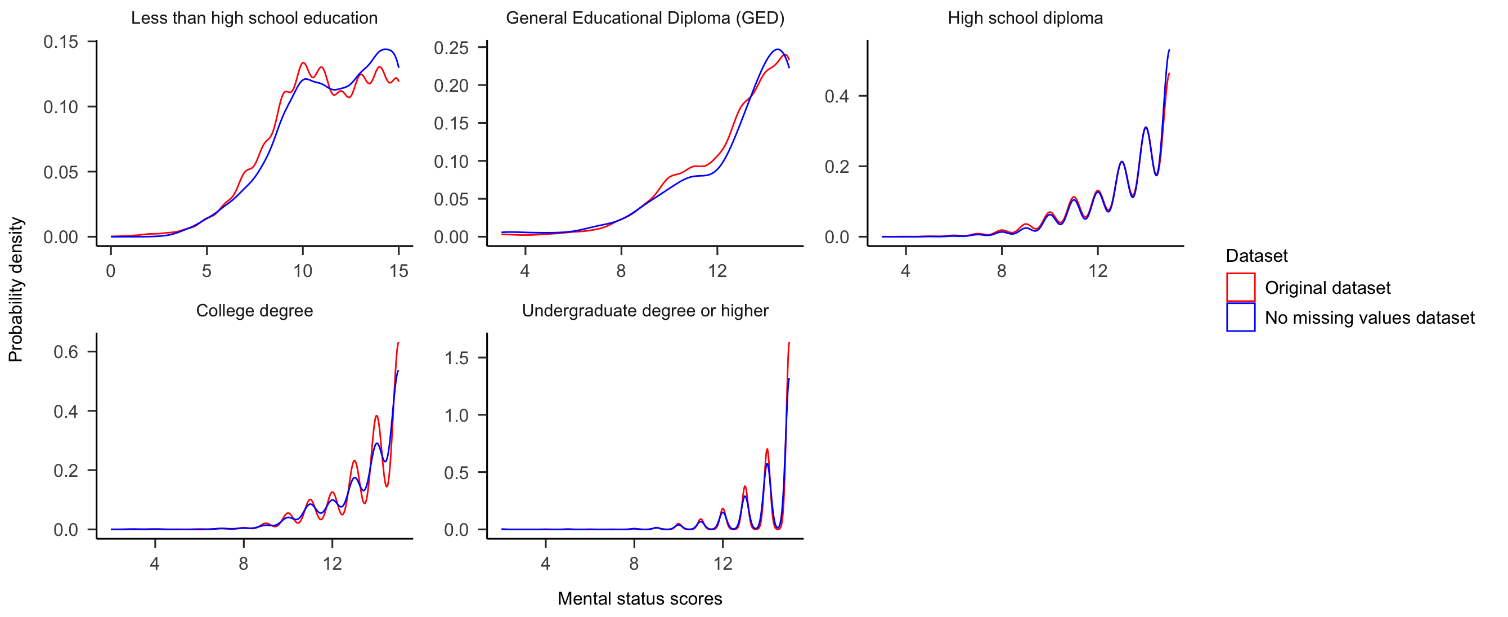
**

**Supplementary Figure 8.** Educational Impact on Mental Status (Y2) in Original and No Missing Data Sets

**
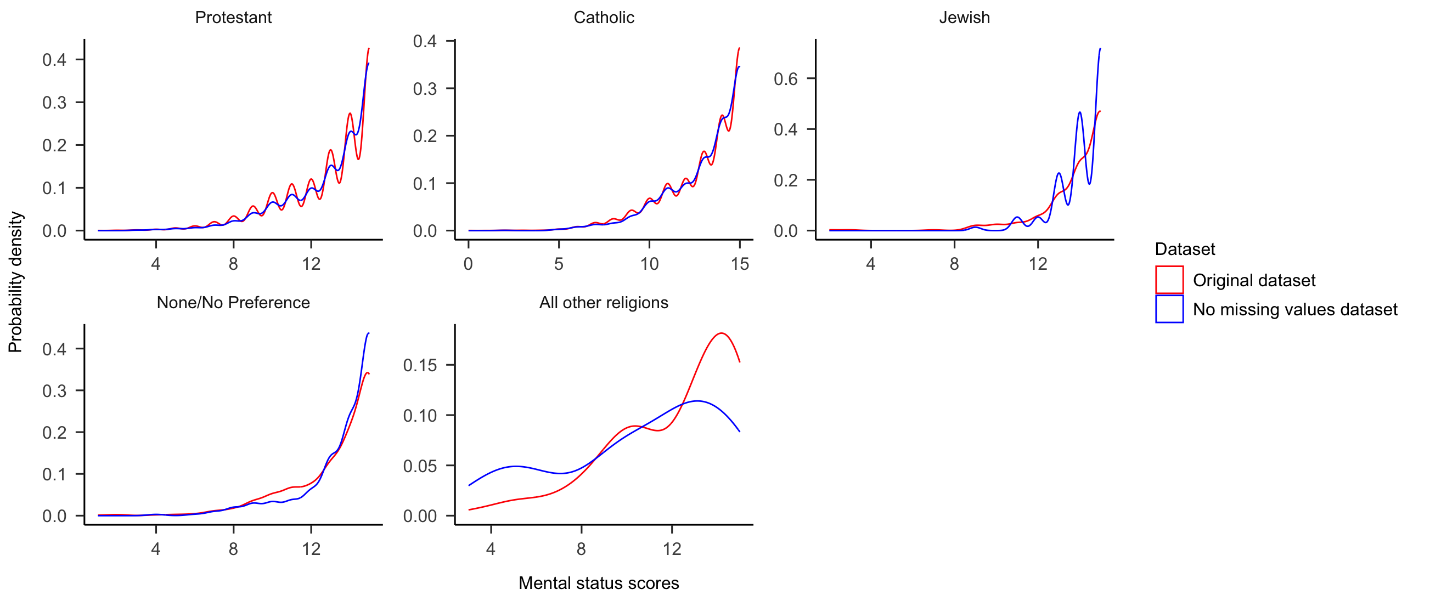
**

**Supplementary Figure 9.** Distribution of Mental Status (Y2) by Religion in Original and No Missing Data Subsets

*
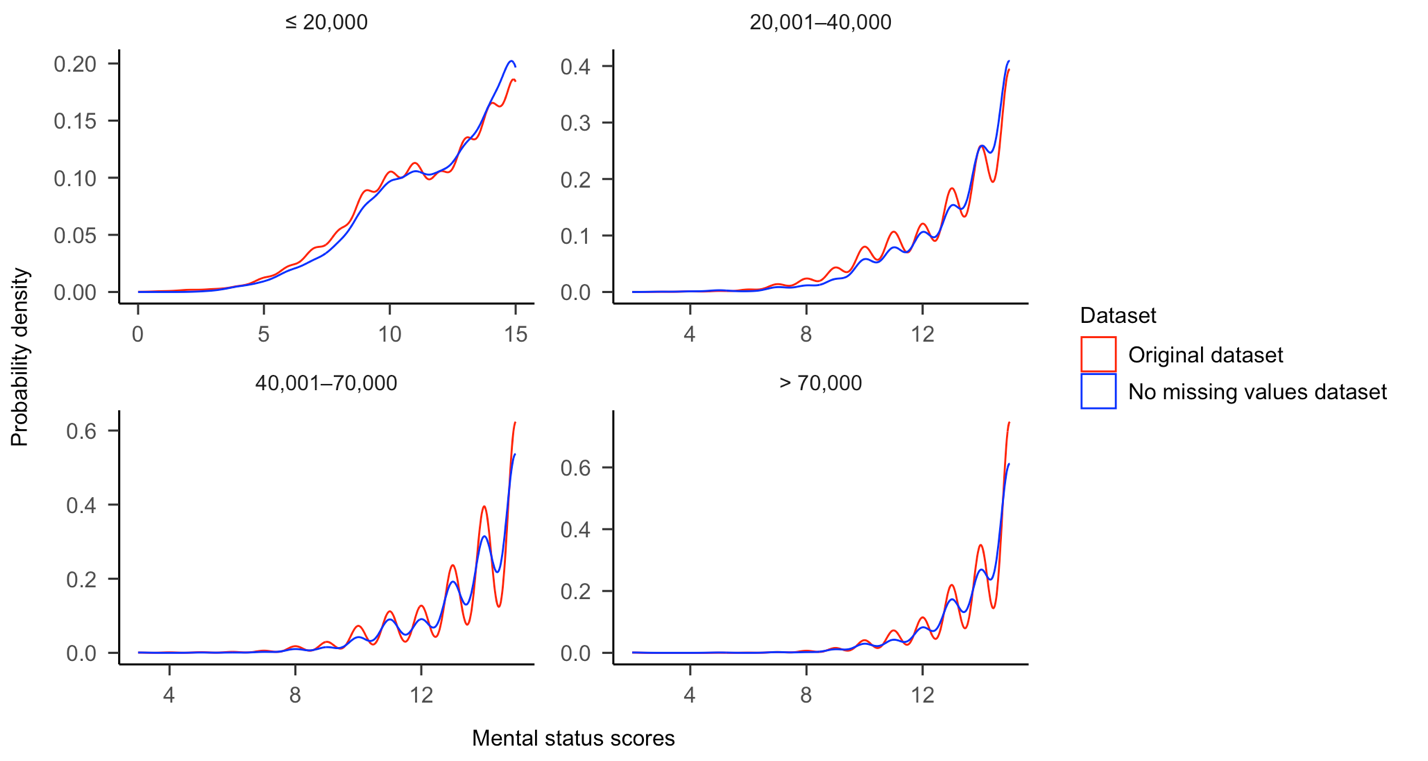
*

**Supplementary Figure 10.** Distribution of Mental Status (Y2) by Income Level in Original and No Missing Data Subsets

# Descriptive Statistics for Dependent Variables

This appendix includes figures showing the trends of the dependent variables, Episodic Memory (Y1) and Mental Status (Y2), over time. The first part presents plots of mean values at each time point to illustrate overall trends in two outcomes. The second part displays mean values over time, grouped by three moderator combinations. These plots highlight different trajectories based on moderator combinations and support the intersectionality approach used in this study.

## Supplementary Figures

**
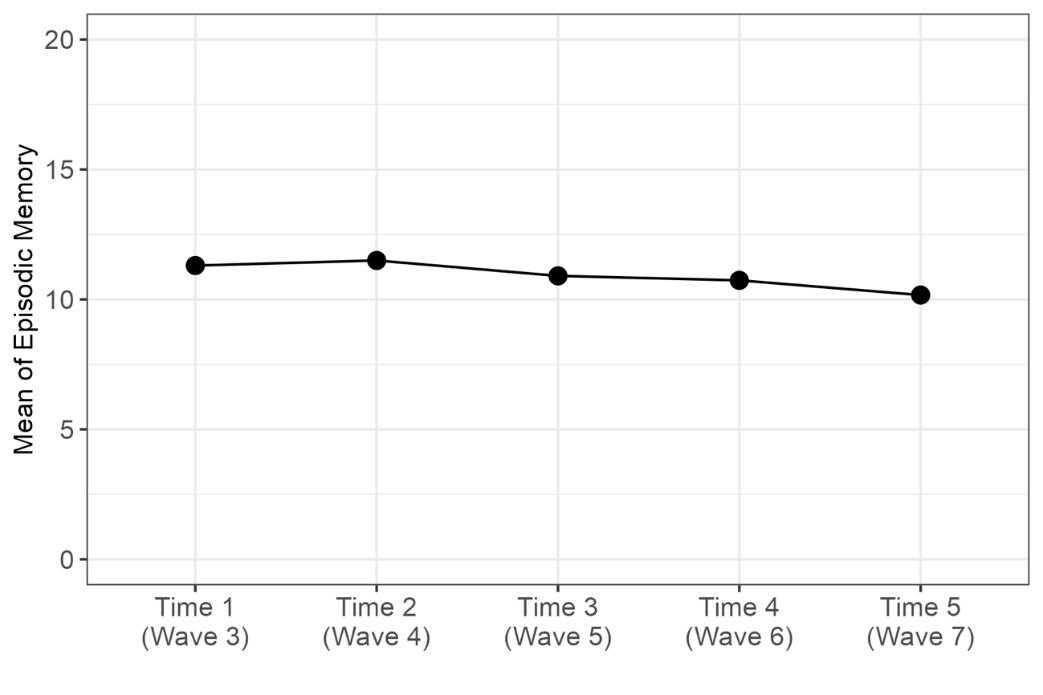
**

**Supplementary Figure 1.** Mean of Episodic Memory (Y1) Over Time

**
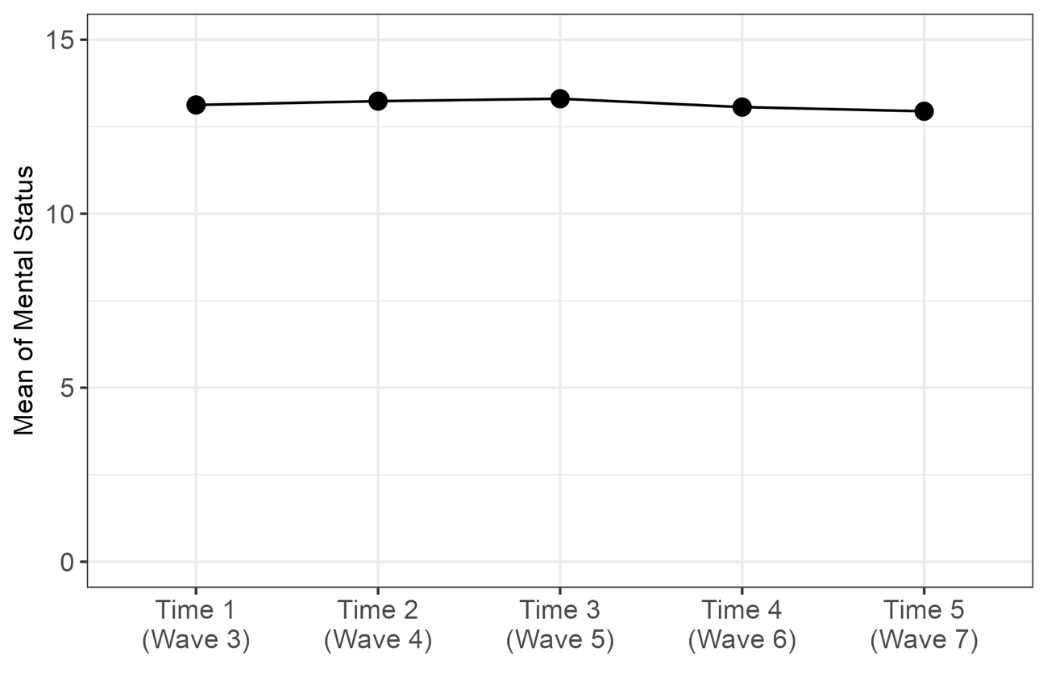
**

**Supplementary Figure 2.** Mean of Mental Status (Y2) Over Time

**
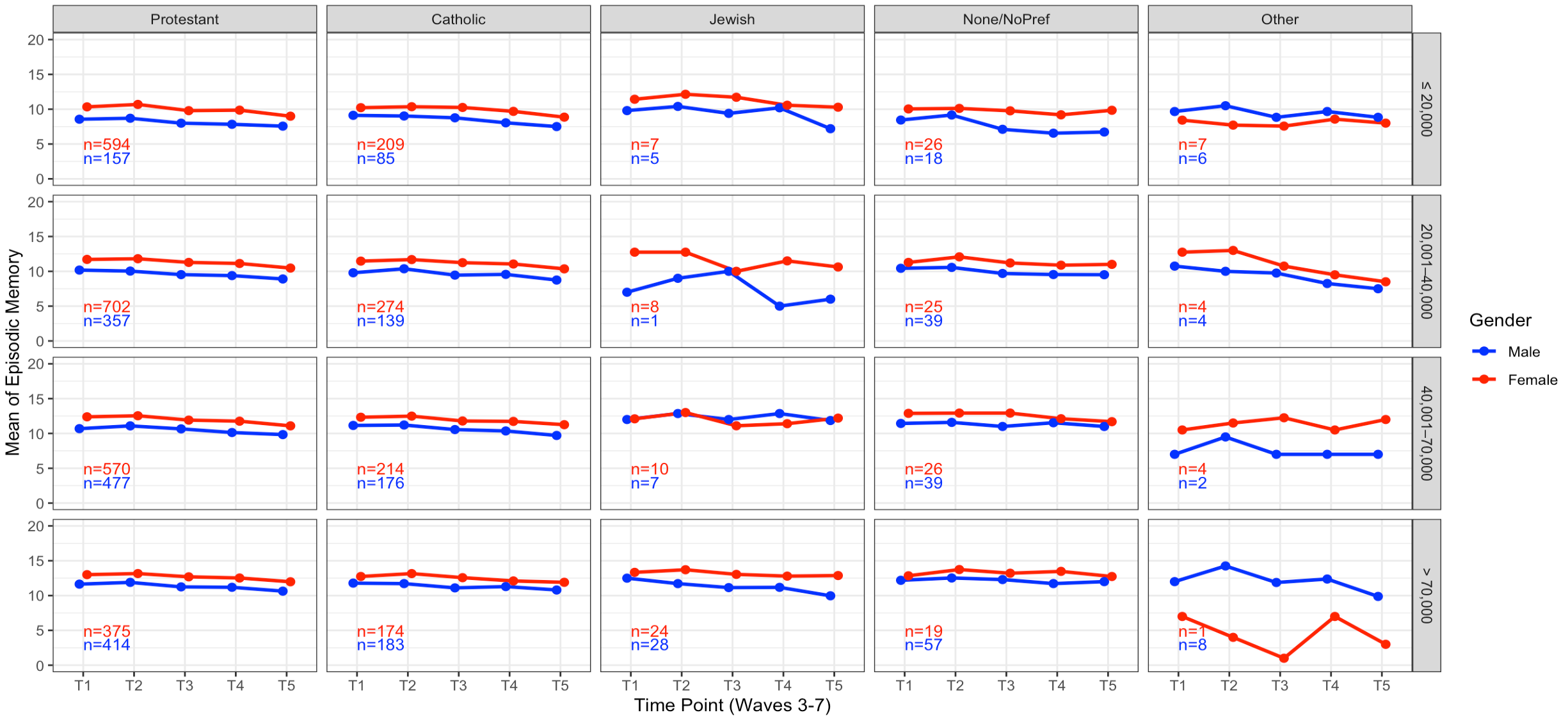
**

**Supplementary Figure 3.** Mean of Episodic Memory by Gender, Religion, and Total Income across Time Points
*Note.* The categorization of Total Income in the row is based on the average of Total Income across five time waves, ensuring that individuals remain in the same panel across time, as Total Income can change over time.


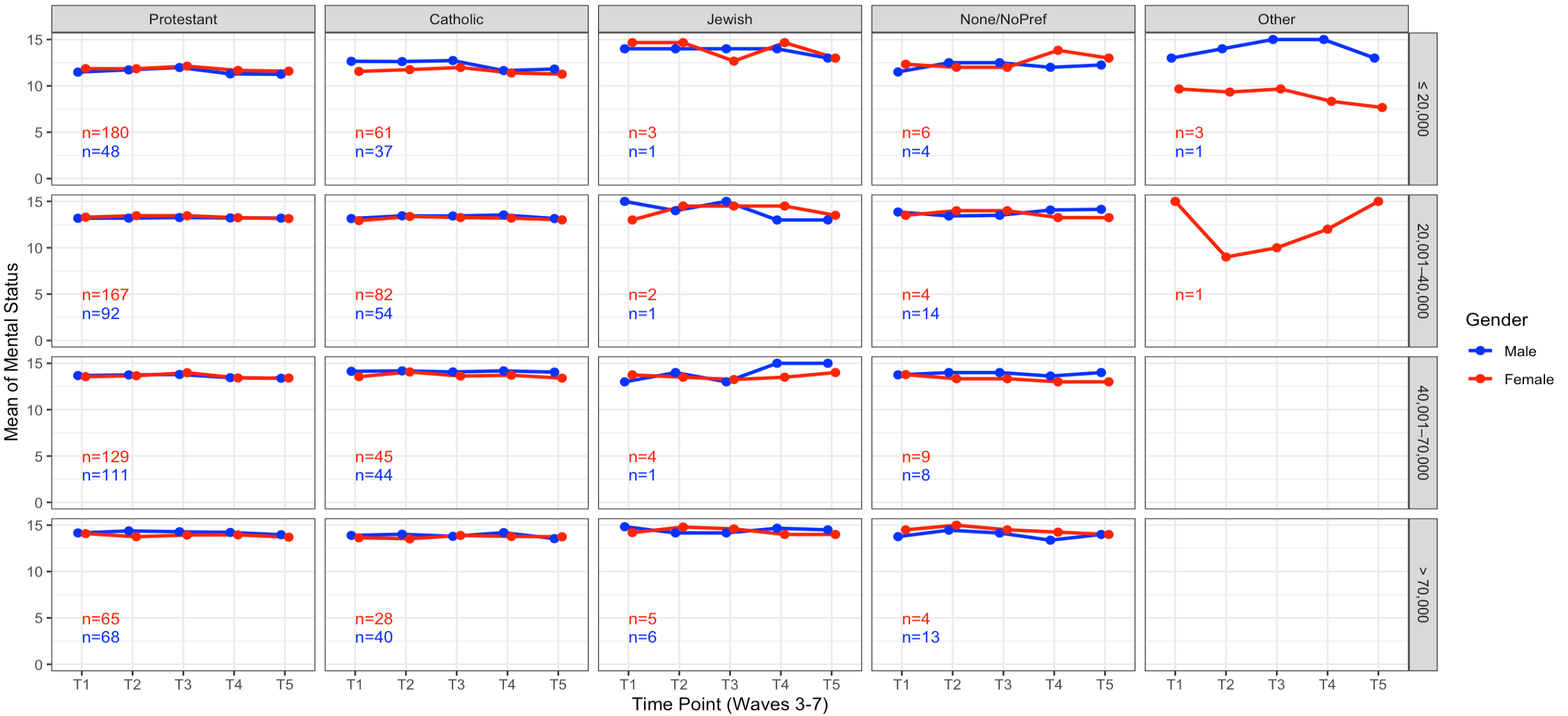


**Supplementary Figure 4.** Mean of Mental Status by Gender, Religion, and Total Income across Time Points
*Note.* The categorization of Total Income in the row is based on the average of Total Income across five time waves, ensuring that individuals remain in the same panel across time, as Total Income can change over time. The absence of line(s) in the panel indicates that no participants fall within that specific intersection of variables (e.g., religion = Other, and Total Income = ‘40,001-70,000’ or ‘>70,000’).


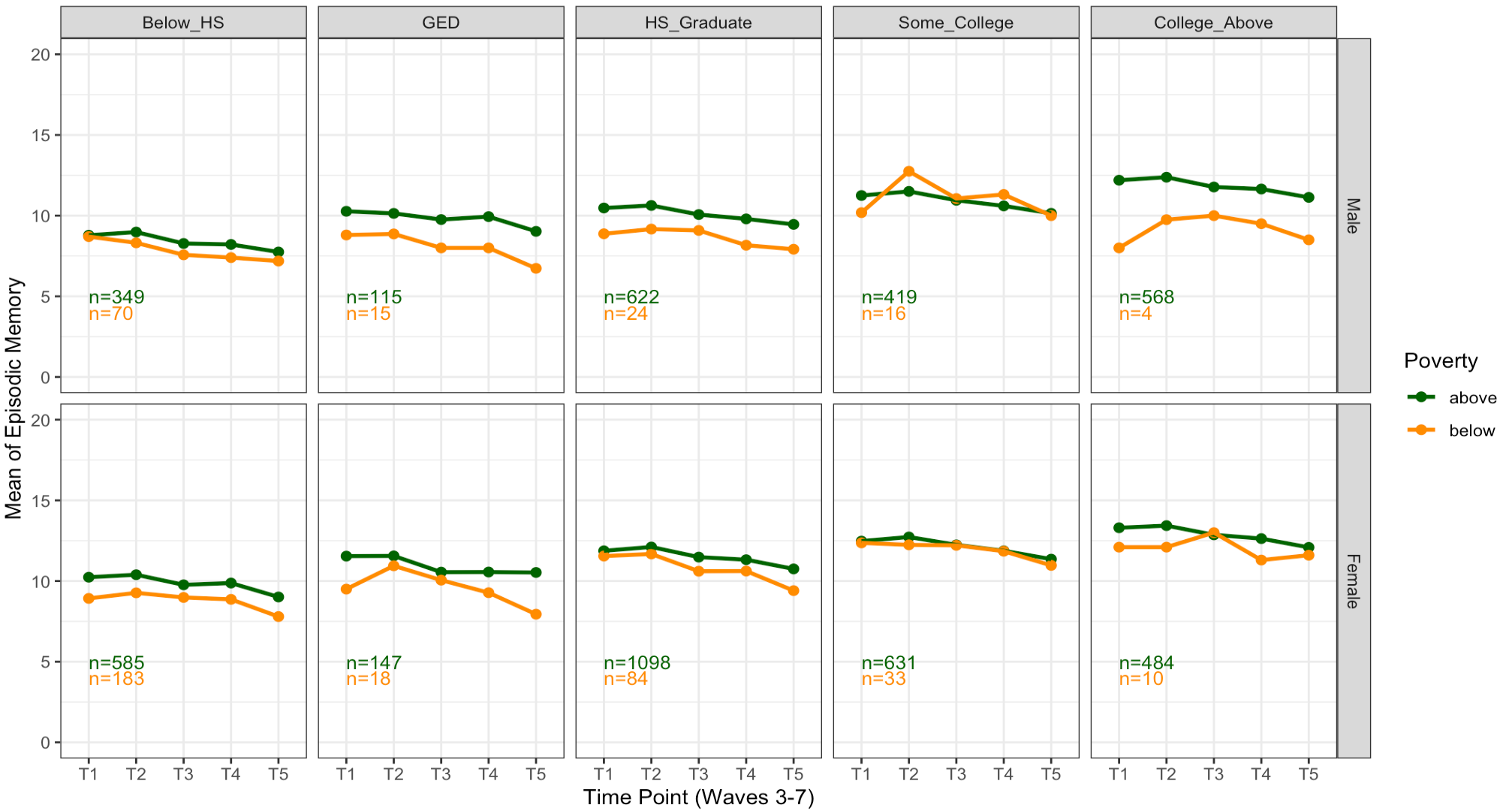


**Supplementary Figure 5.** Mean of Episodic Memory by Gender, Race, and Poverty Status across Time Points
*Note.* In this visualization, the Poverty variable indicates the poverty status at the first time point, allowing individuals to stay in the same panel across time, as poverty status can change over time.


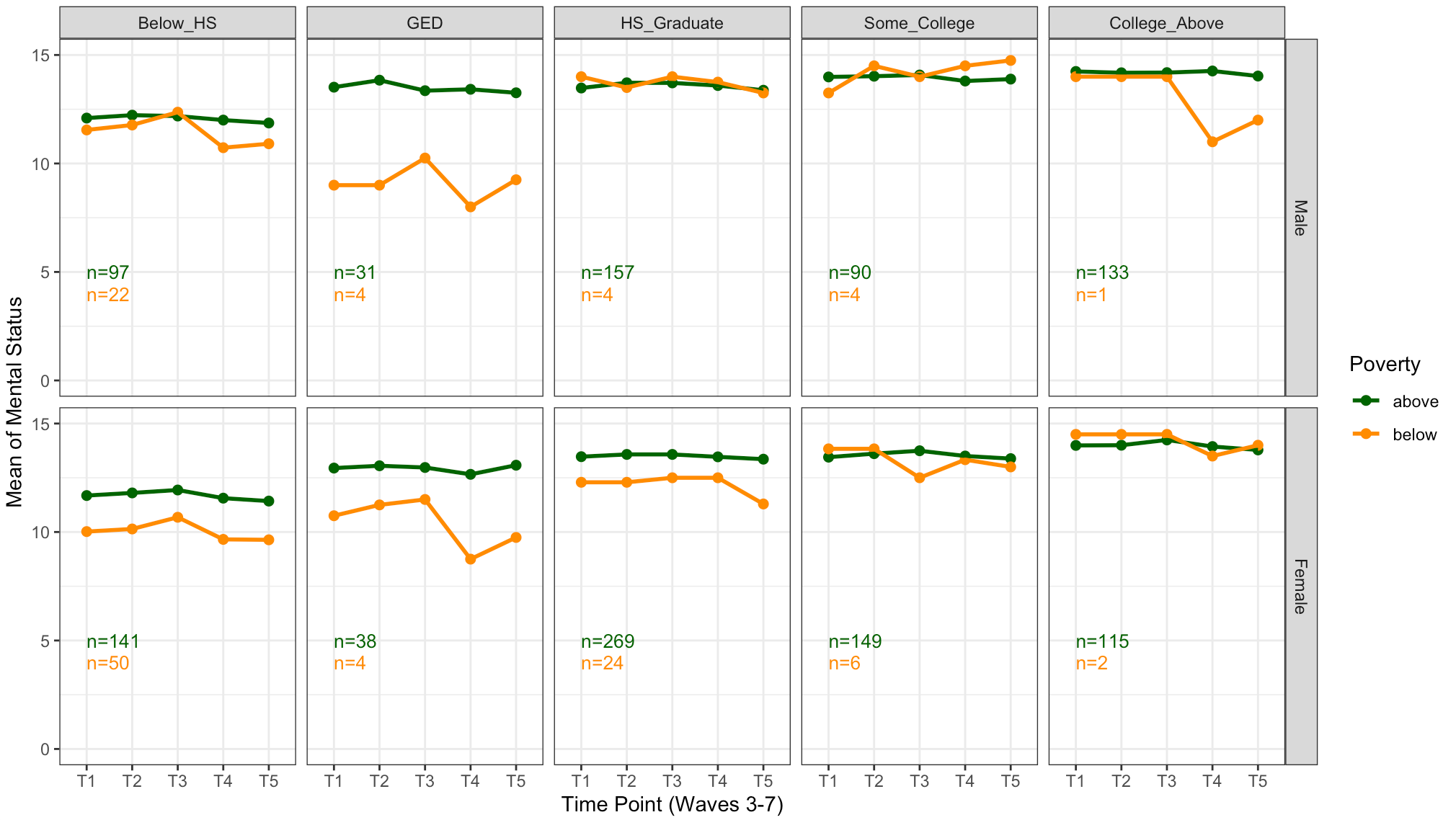


**Supplementary Figure 6.** Mean of Mental Status by Gender, Race, and Poverty Status across Time Points
*Note.* In this visualization, the Poverty variable indicates the poverty status at the first time point, allowing individuals to stay in the same panel across time, as poverty status can change over time.

# Example Code with Annotations

This supplementary material provides the R code used for data preprocessing, baseline model fitting, and GLMM-tree analysis described in the manuscript. The script includes all steps from loading and preparing the data to fitting and visualizing the models.

#############################################

# Step 1. Load packages

#############################################

library(tidyverse)

library(glmertree)

library(lme4)

#############################################

# Step 2. Load and Preprocess Datasets

#############################################

# Variable name mapping (manuscript variable name -> code variable):

# episodic_memory = Total_WRimp (HRS imputed word-recall score, Y1)

# mental_status = Total_MSimp (HRS imputed mental-status score, Y2)

# age_centered_within = Age_cw1 (person-mean-centered age; shown as Age_cw1 in Figures 3 and 4)

# time = Time (wave indicator)

# participant_id = HHIDPN (HRS person identifier)

# 1) Load the datasets

epi <- read.csv("Episodic Memory no missing N5475.csv")

ment <- read.csv("Mental Status no missing N1341.csv")

# 2) Preprocess function (unordered factors)

variable_setting <- function(df) {

df %>%

mutate(

Gender = factor(Gender, levels = c("Male", "Female")),

Race = factor(Race, levels = c("White/Caucasian",

"Black/African American",

"Other")),

Religion = factor(Religion, levels = c("Protestant", "Catholic",

"Jewish", "None/NoPref", "Other")),

Poverty = factor(Poverty, levels = c("above", "below")),

Education = factor(ifelse(Education <= 11, '11_or_less', 'more_than_11'),

levels = c('more_than_11', '11_or_less')),

Age_mean1 = ave(Age, HHIDPN, FUN = mean),

Age_cw1 = Age - Age_mean1 # center Age variable

)

}

# 3) Apply to both datasets

ment <- variable_setting(ment)

epi <- variable_setting(epi)

# Check — should all be "factor"

sapply(epi[, c("Gender","Race","Religion","Poverty","Education")], class)

sapply(ment[, c("Gender","Race","Religion","Poverty","Education")], class)

#############################################

# Step 3. Define GLMM-tree helper function

#############################################

glmmtree_analysis <- function(formula, data, model_name, plot_type = c("simple", "combined")) {

plot_type <- match.arg(plot_type)

lt <- lmertree(formula, data = data, maxdepth = 4)

png(paste0(model_name, "_tree.png"),

width = 15, height = 10, units = "in",

res = 600, pointsize = 12)

if (plot_type == "simple") {

plot(lt, type = "simple", which = "tree")

} else {

plot(lt, which = "tree", fitted = "combined")

}

dev.off()

return(lt)

}

#############################################

# Step 4. Baseline LMM

#############################################

# Episodic memory

lmm_epi <- lmer(Total_WRimp ~ Age_cw1 + (1 + Time | HHIDPN), data = epi)

summary(lmm_epi)

# Mental status

lmm_ment <- lmer(Total_MSimp ~ 1 + (1 | HHIDPN), data = ment)

summary(lmm_ment)

#############################################

# Step 5. Run GLMM Tree Analysis

#############################################

# Episodic Memory model — simple tree plot

glmmtree_analysis(

Total_WRimp ~ Age_cw1 | (1 + Time | HHIDPN) |

Gender + Race + Education + Religion + Total_Income + Poverty,

data = epi, model_name = "episodic_memory",

plot_type = "simple"

)

# Mental Status model — tree plot

glmmtree_analysis(

Total_MSimp ~ 1 | (1 | HHIDPN) |

Gender + Race + Education + Religion + Total_Income + Poverty,

data = ment, model_name = "mental_status",

plot_type = "combined"

)
